# Author Correction: A molecular timescale for eukaryote evolution with implications for the origin of red algal-derived plastids

**DOI:** 10.1038/s41467-021-23847-w

**Published:** 2021-06-07

**Authors:** Jürgen F. H. Strassert, Iker Irisarri, Tom A. Williams, Fabien Burki

**Affiliations:** 1 Department of Organismal Biology, Program in Systematic Biology, Uppsala University, Uppsala, Sweden; 2 Department of Biodiversity and Evolutionary Biology, Museo Nacional de Ciencias Naturales (MNCN-CSIC), Madrid, Spain; 3 School of Biological Sciences, University of Bristol, Life Sciences Building, Bristol, UK; 4 Science for Life Laboratory, Uppsala University, Uppsala, Sweden; 5 Department of Ecosystem Research, Leibniz Institute of Freshwater Ecology and Inland Fisheries, Berlin, Germany; 6 Department of Applied Bioinformatics, Institute for Microbiology and Genetics, University of Göttingen, and Campus Institute Data Science (CIDAS), Göttingen, Germany

**Keywords:** Molecular evolution, Phylogenetics, Symbiosis, Chloroplasts

Correction to: *Nature Communications* 10.1038/s41467-021-22044-z, published online 25 March 2021.

The original version of this Article contained an error in Fig. 3, in which the bar describing the group Obazoa omitted *Thecamonas trahens* and *Pygsuia biforma*. The correct version of Fig. 3 is:
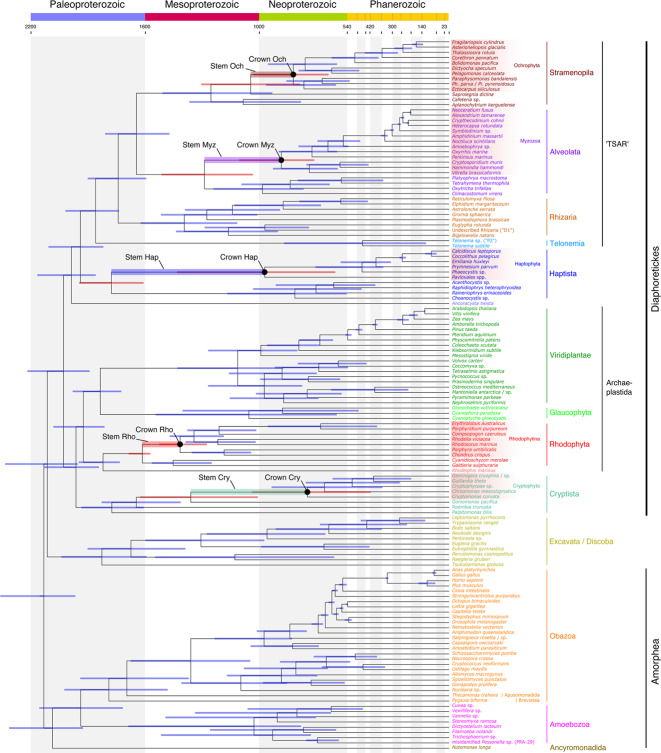


which replaces the previous incorrect version:
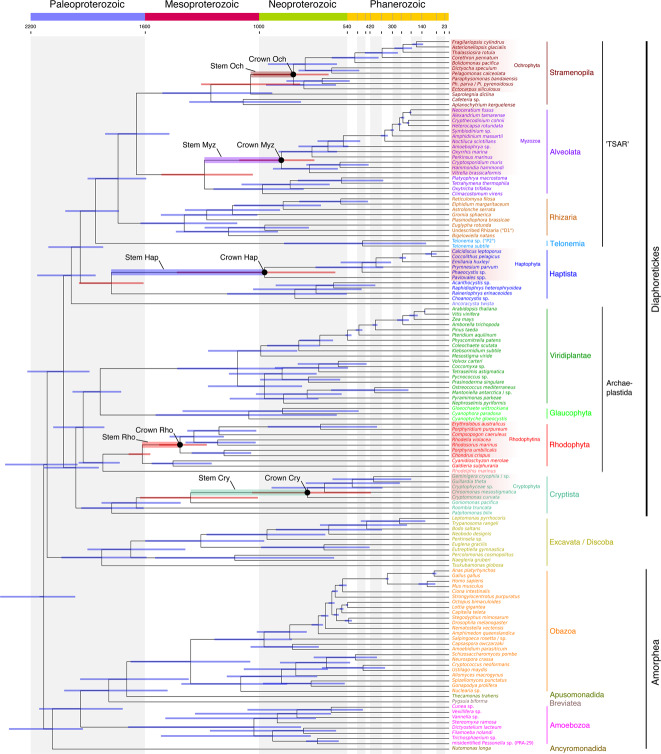


The original version of this Article contained an error in the Introduction, which incorrectly read ‘Plastids (= chloroplasts) are organelles that allow eukaryotes to perform oxygenic photosynthesis.’ The correct version states ‘e.g.’ in place of ‘=’.

These have been corrected in both the PDF and HTML versions of the Article.

