# Supplementary material for: A molecular timescale for eukaryote evolution with implications for the origin of red algal-derived plastids
Source: Nat Commun. 2021 Mar 25;12:1879. doi: 10.1038/s41467-021-22044-z (PMC7994803; doi:10.1038/s41467-021-22044-z)
Supplement: Supplementary file 3 — Description of Additional Supplementary Files [file 41467_2021_22044_MOESM3_ESM.pdf]

Description of Additional Supplementary Files

File Name: Supplementary Data 1
Description: Molecular clock results and violin plots

File Name: Supplementary Data 2
Description: Sequence data, single gene trees, and taxonomy files
