# Supplementary material for: A molecular timescale for eukaryote evolution with implications for the origin of red algal-derived plastids
Source: Nat Commun. 2021 Mar 25;12:1879. doi: 10.1038/s41467-021-22044-z (PMC7994803; doi:10.1038/s41467-021-22044-z)
Supplement: Supplementary file 4 — Supplementary Data 1 [file 41467_2021_22044_MOESM4_ESM.zip › Supplementary_Data_1/Violin_plots_all_nodes.pdf]

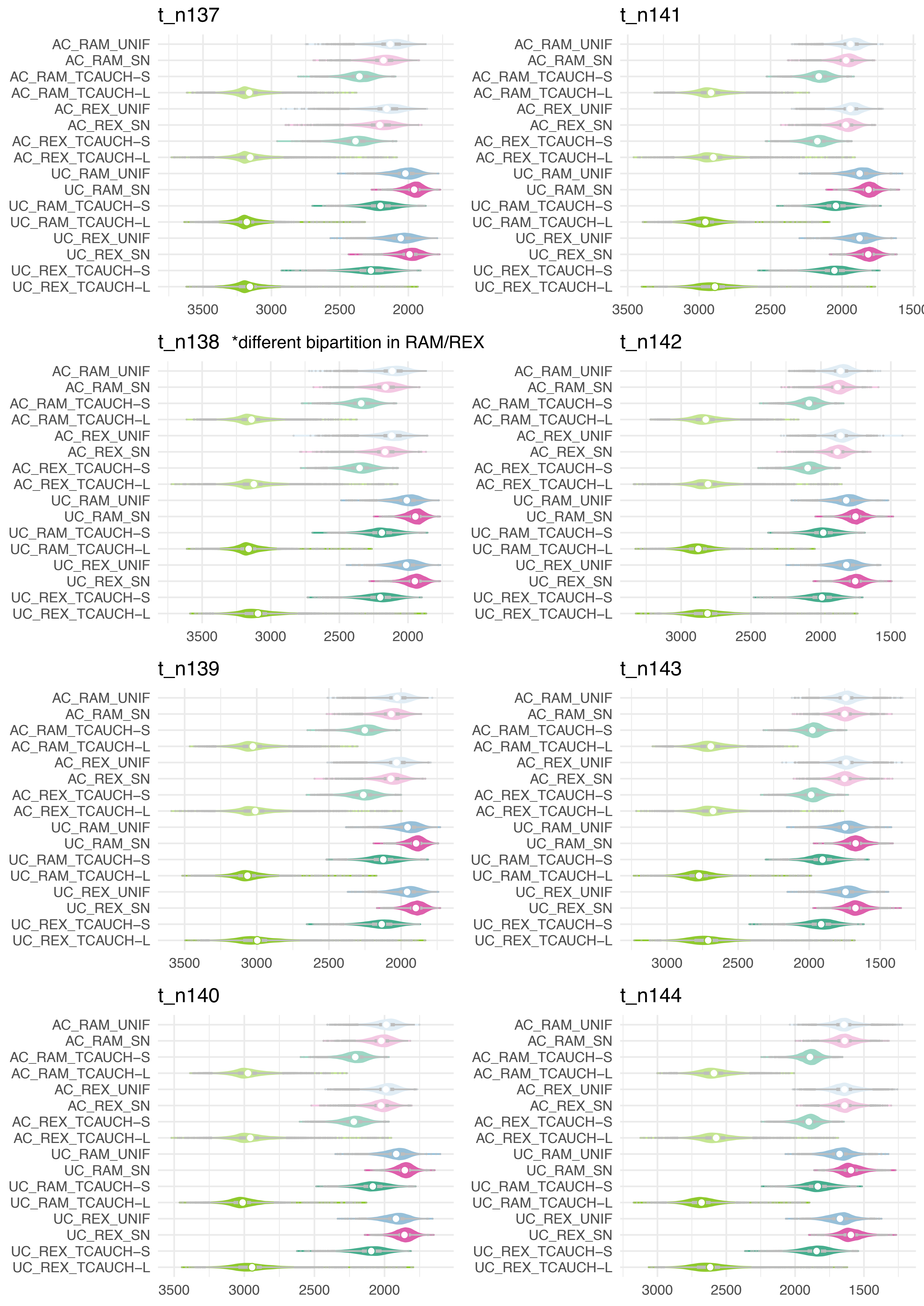
t_n137
AC_RAM_UNIF
AC_RAM_SN
AC_RAM_TCAUCH–S
AC_RAM_TCAUCH–L
AC_REX_UNIF
AC_REX_SN
AC_REX_TCAUCH–S
AC_REX_TCAUCH–L
UC_RAM_UNIF
UC_RAM_SN
UC_RAM_TCAUCH–S
UC_RAM_TCAUCH–L
UC_REX_UNIF
UC_REX_SN
UC_REX_TCAUCH–S
UC_REX_TCAUCH–L
3500
3000
2500
2000
t_n141
AC_RAM_UNIF
AC_RAM_SN
AC_RAM_TCAUCH–S
AC_RAM_TCAUCH–L
AC_REX_UNIF
AC_REX_SN
AC_REX_TCAUCH–S
AC_REX_TCAUCH–L
UC_RAM_UNIF
UC_RAM_SN
UC_RAM_TCAUCH–S
UC_RAM_TCAUCH–L
UC_REX_UNIF
UC_REX_SN
UC_REX_TCAUCH–S
UC_REX_TCAUCH–L
3500
3000
2500
2000
150
t_n138 *different bipartition in RAM/REX
AC_RAM_UNIF
AC_RAM_SN
AC_RAM_TCAUCH–S
AC_RAM_TCAUCH–L
AC_REX_UNIF
AC_REX_SN
AC_REX_TCAUCH–S
AC_REX_TCAUCH–L
UC_RAM_UNIF
UC_RAM_SN
UC_RAM_TCAUCH–S
UC_RAM_TCAUCH–L
UC_REX_UNIF
UC_REX_SN
UC_REX_TCAUCH–S
UC_REX_TCAUCH–L
3500
3000
2500
2000
t_n142
AC_RAM_UNIF
AC_RAM_SN
AC_RAM_TCAUCH–S
AC_RAM_TCAUCH–L
AC_REX_UNIF
AC_REX_SN
AC_REX_TCAUCH–S
AC_REX_TCAUCH–L
UC_RAM_UNIF
UC_RAM_SN
UC_RAM_TCAUCH–S
UC_RAM_TCAUCH–L
UC_REX_UNIF
UC_REX_SN
UC_REX_TCAUCH–S
UC_REX_TCAUCH–L
3000
2500
2000
1500
t_n139
AC_RAM_UNIF
AC_RAM_SN
AC_RAM_TCAUCH–S
AC_RAM_TCAUCH–L
AC_REX_UNIF
AC_REX_SN
AC_REX_TCAUCH–S
AC_REX_TCAUCH–L
UC_RAM_UNIF
UC_RAM_SN
UC_RAM_TCAUCH–S
UC_RAM_TCAUCH–L
UC_REX_UNIF
UC_REX_SN
UC_REX_TCAUCH–S
UC_REX_TCAUCH–L
3500
3000
2500
2000
t_n143
AC_RAM_UNIF
AC_RAM_SN
AC_RAM_TCAUCH–S
AC_RAM_TCAUCH–L
AC_REX_UNIF
AC_REX_SN
AC_REX_TCAUCH–S
AC_REX_TCAUCH–L
UC_RAM_UNIF
UC_RAM_SN
UC_RAM_TCAUCH–S
UC_RAM_TCAUCH–L
UC_REX_UNIF
UC_REX_SN
UC_REX_TCAUCH–S
UC_REX_TCAUCH–L
3000
2500
2000
1500
t_n140
AC_RAM_UNIF
AC_RAM_SN
AC_RAM_TCAUCH–S
AC_RAM_TCAUCH–L
AC_REX_UNIF
AC_REX_SN
AC_REX_TCAUCH–S
AC_REX_TCAUCH–L
UC_RAM_UNIF
UC_RAM_SN
UC_RAM_TCAUCH–S
UC_RAM_TCAUCH–L
UC_REX_UNIF
UC_REX_SN
UC_REX_TCAUCH–S
UC_REX_TCAUCH–L
3500
3000
2500
2000
t_n144
AC_RAM_UNIF
AC_RAM_SN
AC_RAM_TCAUCH–S
AC_RAM_TCAUCH–L
AC_REX_UNIF
AC_REX_SN
AC_REX_TCAUCH–S
AC_REX_TCAUCH–L
UC_RAM_UNIF
UC_RAM_SN
UC_RAM_TCAUCH–S
UC_RAM_TCAUCH–L
UC_REX_UNIF
UC_REX_SN
UC_REX_TCAUCH–S
UC_REX_TCAUCH–L
3000
2500
2000
1500

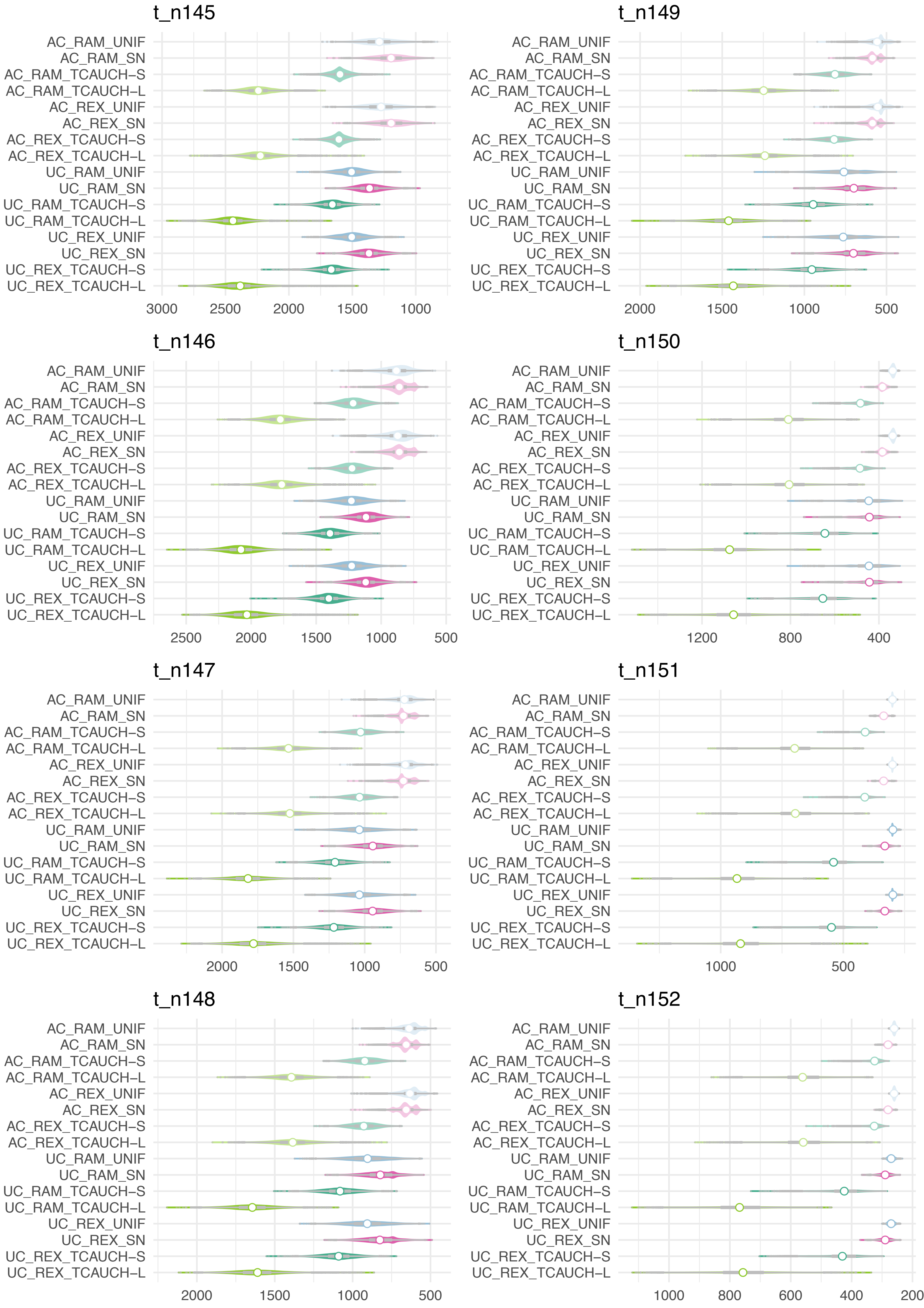

t_n145
AC_RAM_UNIF
AC_RAM_SN
AC_RAM_TCAUCH–S
AC_RAM_TCAUCH–L
AC_REX_UNIF
AC_REX_SN
AC_REX_TCAUCH–S
AC_REX_TCAUCH–L
UC_RAM_UNIF
UC_RAM_SN
UC_RAM_TCAUCH–S
UC_RAM_TCAUCH–L
UC_REX_UNIF
UC_REX_SN
UC_REX_TCAUCH–S
UC_REX_TCAUCH–L
3000
2500
2000
1500
1000
t_n149
2000
1500
1000
500
t_n146
2500
2000
1500
1000
500
t_n150
1200
800
400
t_n147
2000
1500
1000
500
t_n151
1000
500
t_n148
2000
1500
1000
500
t_n152
1000
800
600
400
200

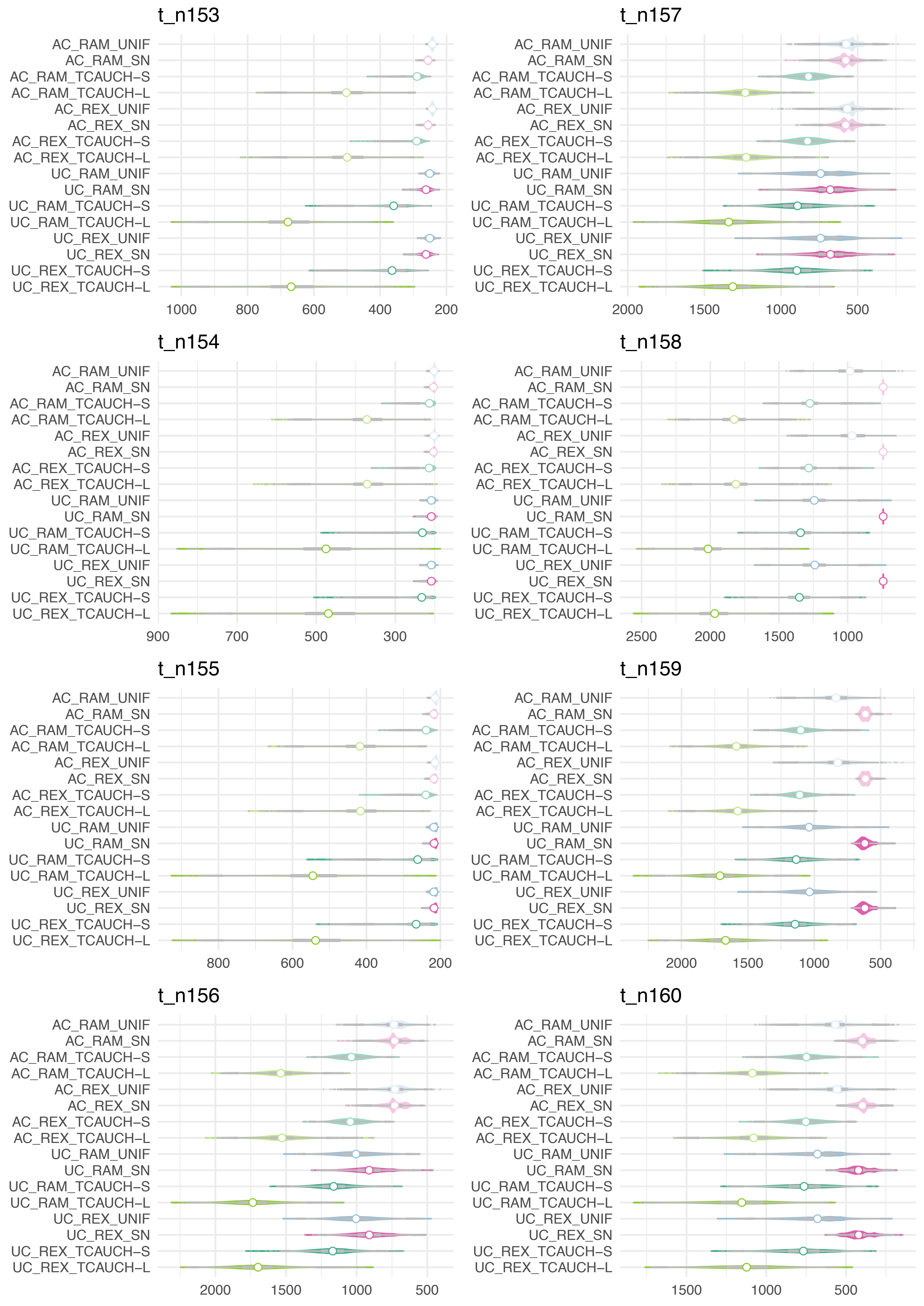
t_n153
AC_RAM_UNIF
AC_RAM_SN
AC_RAM_TCAUCH-S
AC_RAM_TCAUCH-L
AC_REX_UNIF
AC_REX_SN
AC_REX_TCAUCH-S
AC_REX_TCAUCH-L
UC_RAM_UNIF
UC_RAM_SN
UC_RAM_TCAUCH-S
UC_RAM_TCAUCH-L
UC_REX_UNIF
UC_REX_SN
UC_REX_TCAUCH-S
UC_REX_TCAUCH-L
1000
800
600
400
200
t_n157
AC_RAM_UNIF
AC_RAM_SN
AC_RAM_TCAUCH-S
AC_RAM_TCAUCH-L
AC_REX_UNIF
AC_REX_SN
AC_REX_TCAUCH-S
AC_REX_TCAUCH-L
UC_RAM_UNIF
UC_RAM_SN
UC_RAM_TCAUCH-S
UC_RAM_TCAUCH-L
UC_REX_UNIF
UC_REX_SN
UC_REX_TCAUCH-S
UC_REX_TCAUCH-L
2000
1500
1000
500
t_n154
AC_RAM_UNIF
AC_RAM_SN
AC_RAM_TCAUCH-S
AC_RAM_TCAUCH-L
AC_REX_UNIF
AC_REX_SN
AC_REX_TCAUCH-S
AC_REX_TCAUCH-L
UC_RAM_UNIF
UC_RAM_SN
UC_RAM_TCAUCH-S
UC_RAM_TCAUCH-L
UC_REX_UNIF
UC_REX_SN
UC_REX_TCAUCH-S
UC_REX_TCAUCH-L
900
700
500
300
t_n158
AC_RAM_UNIF
AC_RAM_SN
AC_RAM_TCAUCH-S
AC_RAM_TCAUCH-L
AC_REX_UNIF
AC_REX_SN
AC_REX_TCAUCH-S
AC_REX_TCAUCH-L
UC_RAM_UNIF
UC_RAM_SN
UC_RAM_TCAUCH-S
UC_RAM_TCAUCH-L
UC_REX_UNIF
UC_REX_SN
UC_REX_TCAUCH-S
UC_REX_TCAUCH-L
2500
2000
1500
1000
t_n155
AC_RAM_UNIF
AC_RAM_SN
AC_RAM_TCAUCH-S
AC_RAM_TCAUCH-L
AC_REX_UNIF
AC_REX_SN
AC_REX_TCAUCH-S
AC_REX_TCAUCH-L
UC_RAM_UNIF
UC_RAM_SN
UC_RAM_TCAUCH-S
UC_RAM_TCAUCH-L
UC_REX_UNIF
UC_REX_SN
UC_REX_TCAUCH-S
UC_REX_TCAUCH-L
800
600
400
200
t_n159
AC_RAM_UNIF
AC_RAM_SN
AC_RAM_TCAUCH-S
AC_RAM_TCAUCH-L
AC_REX_UNIF
AC_REX_SN
AC_REX_TCAUCH-S
AC_REX_TCAUCH-L
UC_RAM_UNIF
UC_RAM_SN
UC_RAM_TCAUCH-S
UC_RAM_TCAUCH-L
UC_REX_UNIF
UC_REX_SN
UC_REX_TCAUCH-S
UC_REX_TCAUCH-L
2000
1500
1000
500
t_n156
AC_RAM_UNIF
AC_RAM_SN
AC_RAM_TCAUCH-S
AC_RAM_TCAUCH-L
AC_REX_UNIF
AC_REX_SN
AC_REX_TCAUCH-S
AC_REX_TCAUCH-L
UC_RAM_UNIF
UC_RAM_SN
UC_RAM_TCAUCH-S
UC_RAM_TCAUCH-L
UC_REX_UNIF
UC_REX_SN
UC_REX_TCAUCH-S
UC_REX_TCAUCH-L
2000
1500
1000
500
t_n160
AC_RAM_UNIF
AC_RAM_SN
AC_RAM_TCAUCH-S
AC_RAM_TCAUCH-L
AC_REX_UNIF
AC_REX_SN
AC_REX_TCAUCH-S
AC_REX_TCAUCH-L
UC_RAM_UNIF
UC_RAM_SN
UC_RAM_TCAUCH-S
UC_RAM_TCAUCH-L
UC_REX_UNIF
UC_REX_SN
UC_REX_TCAUCH-S
UC_REX_TCAUCH-L
1500
1000
500

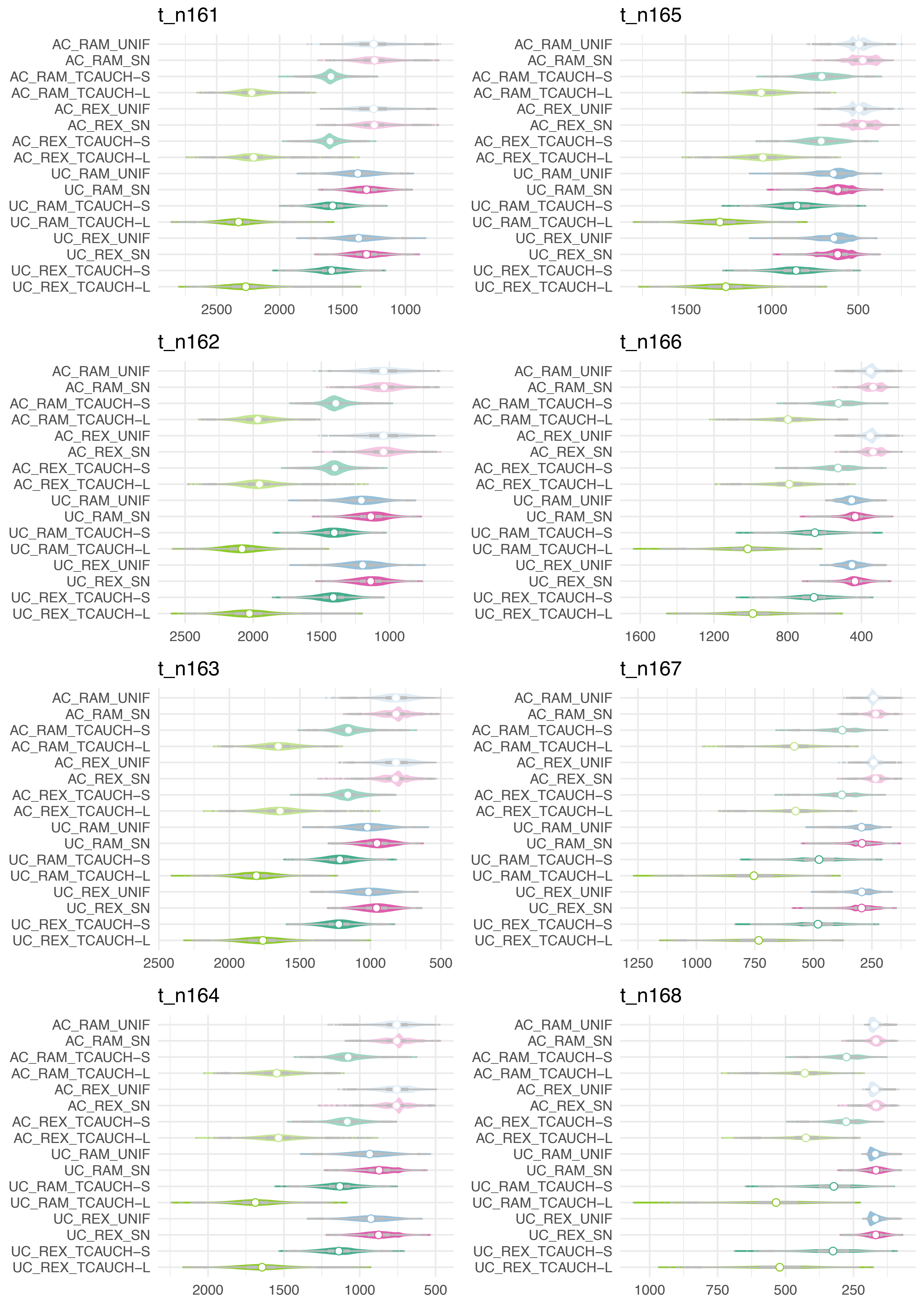

t_n161
AC_RAM_UNIF
AC_RAM_SN
AC_RAM_TCAUCH−S
AC_RAM_TCAUCH−L
AC_REX_UNIF
AC_REX_SN
AC_REX_TCAUCH−S
AC_REX_TCAUCH−L
UC_RAM_UNIF
UC_RAM_SN
UC_RAM_TCAUCH−S
UC_RAM_TCAUCH−L
UC_REX_UNIF
UC_REX_SN
UC_REX_TCAUCH−S
UC_REX_TCAUCH−L
2500
2000
1500
1000
t_n165
1500
1000
500
t_n162
2500
2000
1500
1000
t_n166
1600
1200
800
400
t_n163
2500
2000
1500
1000
500
t_n167
1250
1000
750
500
250
t_n164
2000
1500
1000
500
t_n168
1000
750
500
250

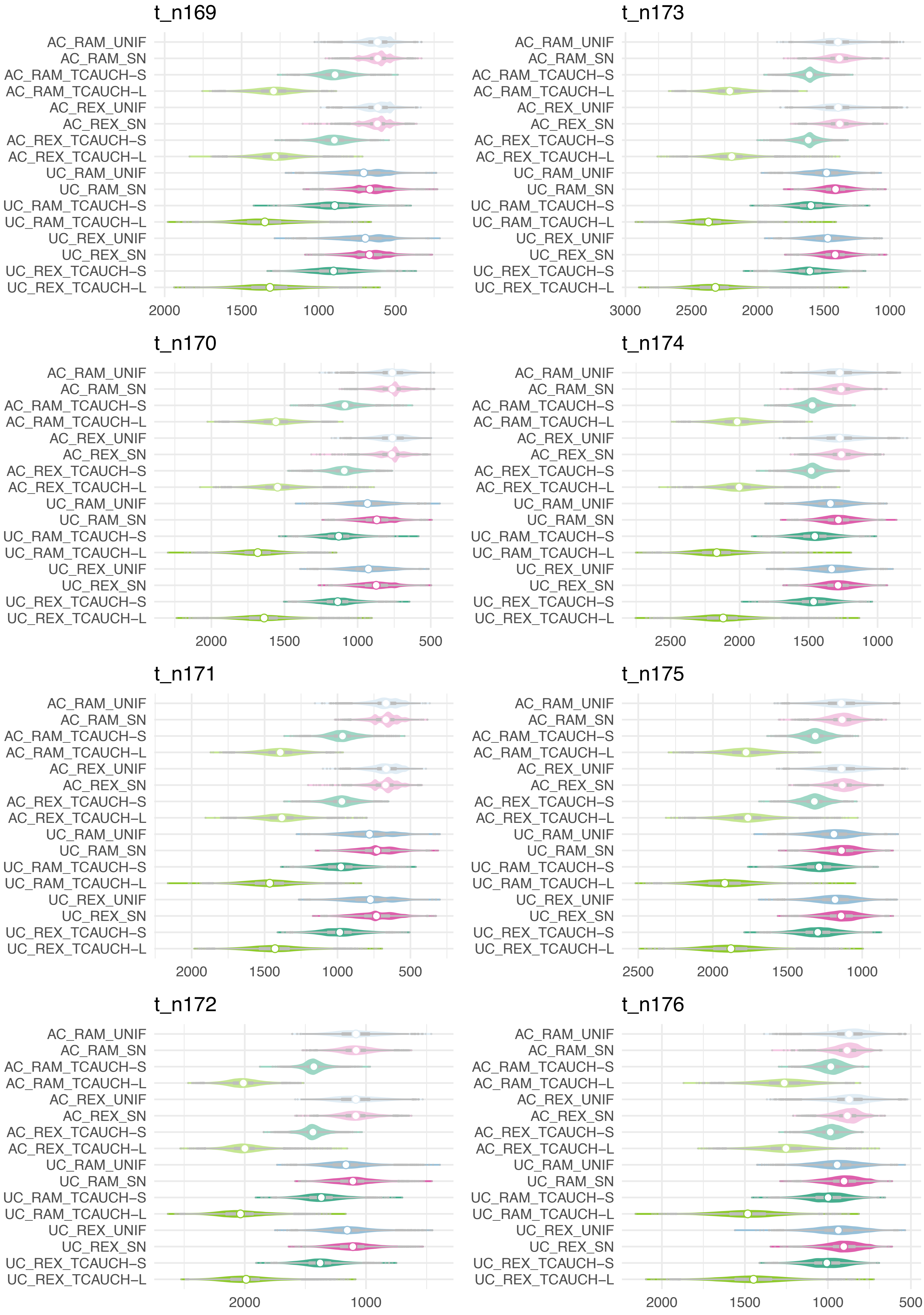
t_n169
AC_RAM_UNIF
AC_RAM_SN
AC_RAM_TCAUCH–S
AC_RAM_TCAUCH–L
AC_REX_UNIF
AC_REX_SN
AC_REX_TCAUCH–S
AC_REX_TCAUCH–L
UC_RAM_UNIF
UC_RAM_SN
UC_RAM_TCAUCH–S
UC_RAM_TCAUCH–L
UC_REX_UNIF
UC_REX_SN
UC_REX_TCAUCH–S
UC_REX_TCAUCH–L
2000
1500
1000
500
t_n173
AC_RAM_UNIF
AC_RAM_SN
AC_RAM_TCAUCH–S
AC_RAM_TCAUCH–L
AC_REX_UNIF
AC_REX_SN
AC_REX_TCAUCH–S
AC_REX_TCAUCH–L
UC_RAM_UNIF
UC_RAM_SN
UC_RAM_TCAUCH–S
UC_RAM_TCAUCH–L
UC_REX_UNIF
UC_REX_SN
UC_REX_TCAUCH–S
UC_REX_TCAUCH–L
3000
2500
2000
1500
1000
t_n170
AC_RAM_UNIF
AC_RAM_SN
AC_RAM_TCAUCH–S
AC_RAM_TCAUCH–L
AC_REX_UNIF
AC_REX_SN
AC_REX_TCAUCH–S
AC_REX_TCAUCH–L
UC_RAM_UNIF
UC_RAM_SN
UC_RAM_TCAUCH–S
UC_RAM_TCAUCH–L
UC_REX_UNIF
UC_REX_SN
UC_REX_TCAUCH–S
UC_REX_TCAUCH–L
2000
1500
1000
500
t_n174
AC_RAM_UNIF
AC_RAM_SN
AC_RAM_TCAUCH–S
AC_RAM_TCAUCH–L
AC_REX_UNIF
AC_REX_SN
AC_REX_TCAUCH–S
AC_REX_TCAUCH–L
UC_RAM_UNIF
UC_RAM_SN
UC_RAM_TCAUCH–S
UC_RAM_TCAUCH–L
UC_REX_UNIF
UC_REX_SN
UC_REX_TCAUCH–S
UC_REX_TCAUCH–L
2500
2000
1500
1000
t_n171
AC_RAM_UNIF
AC_RAM_SN
AC_RAM_TCAUCH–S
AC_RAM_TCAUCH–L
AC_REX_UNIF
AC_REX_SN
AC_REX_TCAUCH–S
AC_REX_TCAUCH–L
UC_RAM_UNIF
UC_RAM_SN
UC_RAM_TCAUCH–S
UC_RAM_TCAUCH–L
UC_REX_UNIF
UC_REX_SN
UC_REX_TCAUCH–S
UC_REX_TCAUCH–L
2000
1500
1000
500
t_n175
AC_RAM_UNIF
AC_RAM_SN
AC_RAM_TCAUCH–S
AC_RAM_TCAUCH–L
AC_REX_UNIF
AC_REX_SN
AC_REX_TCAUCH–S
AC_REX_TCAUCH–L
UC_RAM_UNIF
UC_RAM_SN
UC_RAM_TCAUCH–S
UC_RAM_TCAUCH–L
UC_REX_UNIF
UC_REX_SN
UC_REX_TCAUCH–S
UC_REX_TCAUCH–L
2500
2000
1500
1000
t_n172
AC_RAM_UNIF
AC_RAM_SN
AC_RAM_TCAUCH–S
AC_RAM_TCAUCH–L
AC_REX_UNIF
AC_REX_SN
AC_REX_TCAUCH–S
AC_REX_TCAUCH–L
UC_RAM_UNIF
UC_RAM_SN
UC_RAM_TCAUCH–S
UC_RAM_TCAUCH–L
UC_REX_UNIF
UC_REX_SN
UC_REX_TCAUCH–S
UC_REX_TCAUCH–L
2000
1000
t_n176
AC_RAM_UNIF
AC_RAM_SN
AC_RAM_TCAUCH–S
AC_RAM_TCAUCH–L
AC_REX_UNIF
AC_REX_SN
AC_REX_TCAUCH–S
AC_REX_TCAUCH–L
UC_RAM_UNIF
UC_RAM_SN
UC_RAM_TCAUCH–S
UC_RAM_TCAUCH–L
UC_REX_UNIF
UC_REX_SN
UC_REX_TCAUCH–S
UC_REX_TCAUCH–L
2000
1500
1000
500

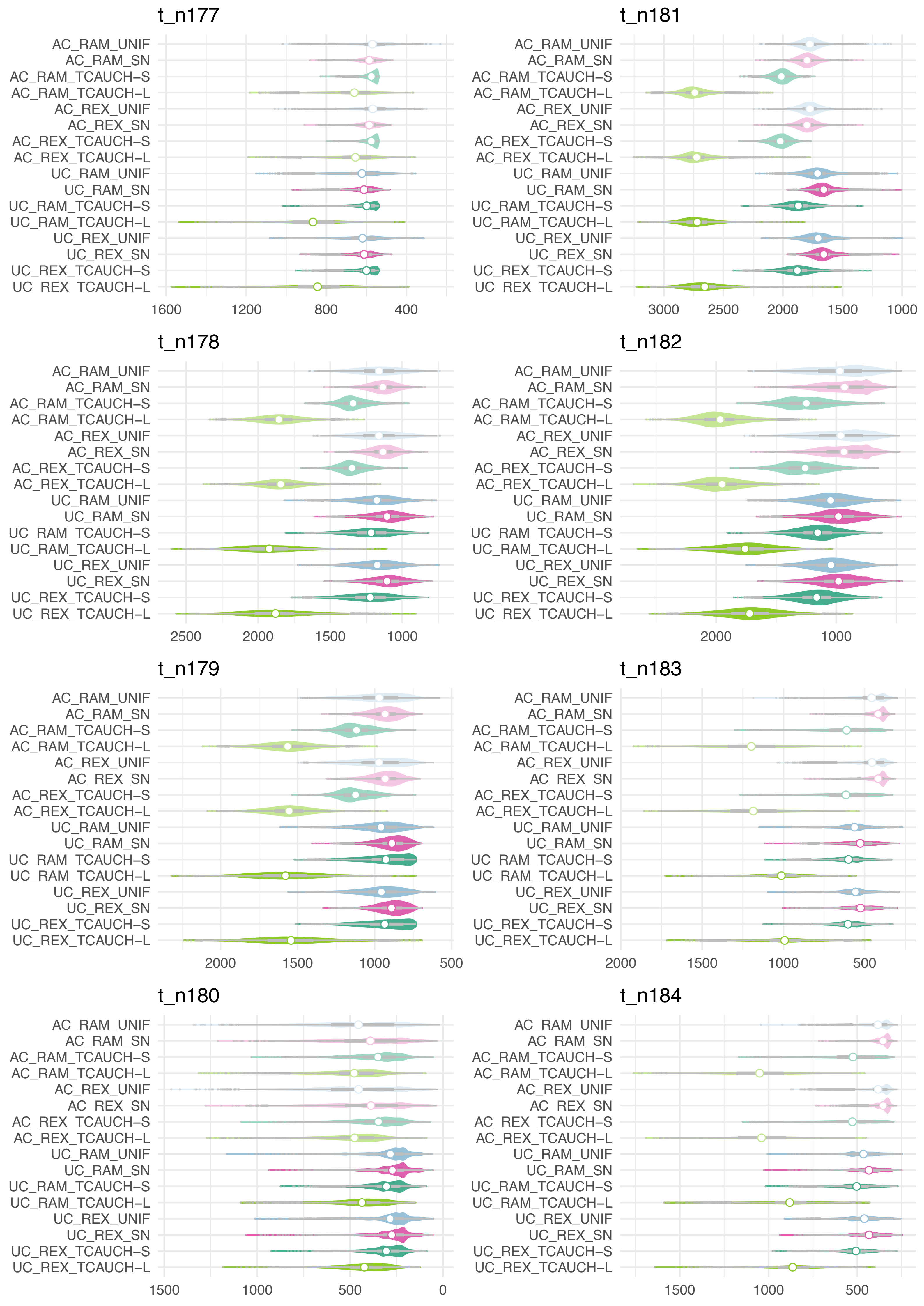
t_n177
AC_RAM_UNIF
AC_RAM_SN
AC_RAM_TCAUCH–S
AC_RAM_TCAUCH–L
AC_REX_UNIF
AC_REX_SN
AC_REX_TCAUCH–S
AC_REX_TCAUCH–L
UC_RAM_UNIF
UC_RAM_SN
UC_RAM_TCAUCH–S
UC_RAM_TCAUCH–L
UC_REX_UNIF
UC_REX_SN
UC_REX_TCAUCH–S
UC_REX_TCAUCH–L
1600
1200
800
400
t_n181
3000
2500
2000
1500
1000
t_n178
2500
2000
1500
1000
t_n182
2000
1000
t_n179
2000
1500
1000
500
t_n183
2000
1500
1000
500
t_n180
1500
1000
500
0
t_n184
1500
1000
500

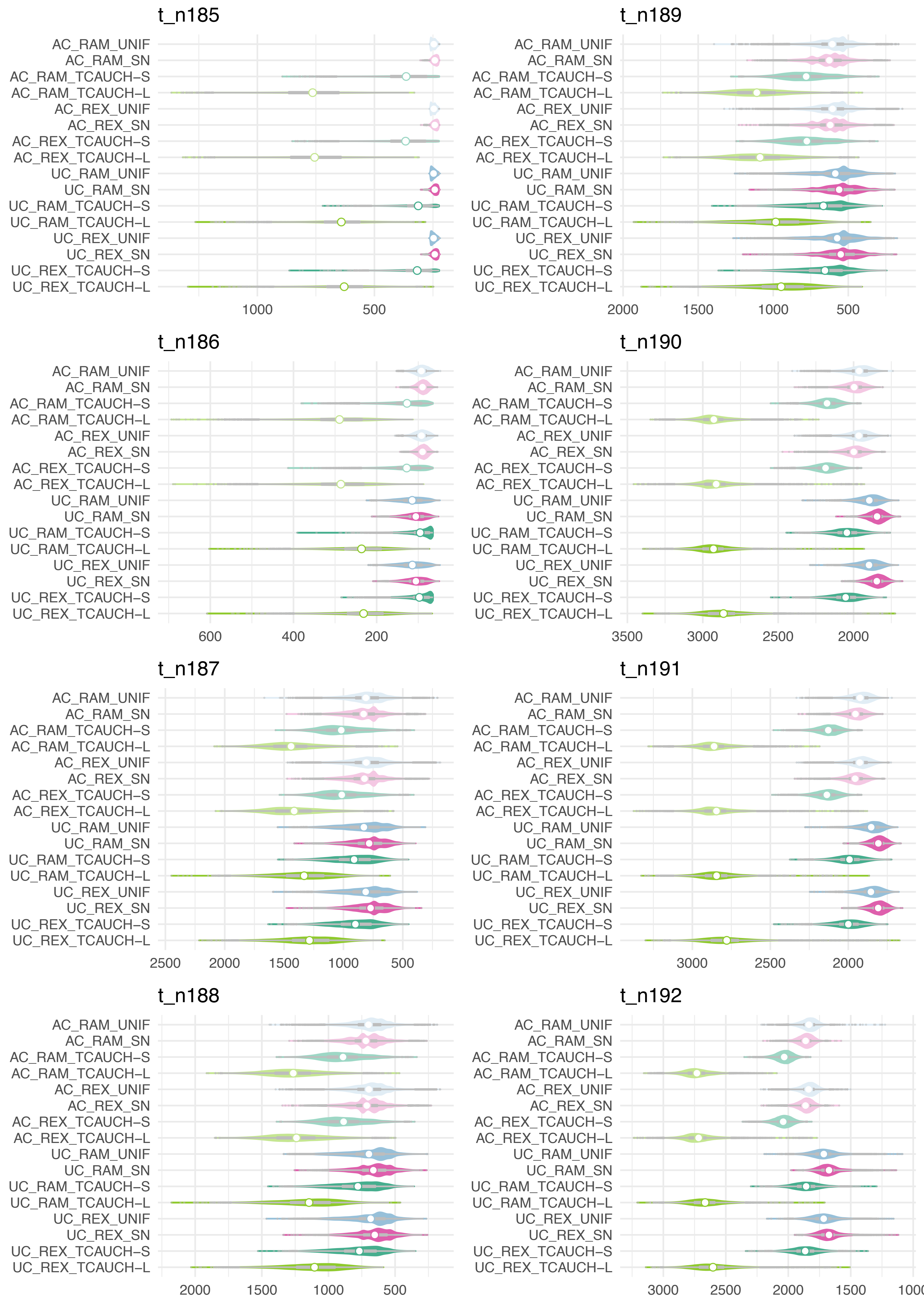
t_n185
AC_RAM_UNIF
AC_RAM_SN
AC_RAM_TCAUCH–S
AC_RAM_TCAUCH–L
AC_REX_UNIF
AC_REX_SN
AC_REX_TCAUCH–S
AC_REX_TCAUCH–L
UC_RAM_UNIF
UC_RAM_SN
UC_RAM_TCAUCH–S
UC_RAM_TCAUCH–L
UC_REX_UNIF
UC_REX_SN
UC_REX_TCAUCH–S
UC_REX_TCAUCH–L
1000
500
t_n189
AC_RAM_UNIF
AC_RAM_SN
AC_RAM_TCAUCH–S
AC_RAM_TCAUCH–L
AC_REX_UNIF
AC_REX_SN
AC_REX_TCAUCH–S
AC_REX_TCAUCH–L
UC_RAM_UNIF
UC_RAM_SN
UC_RAM_TCAUCH–S
UC_RAM_TCAUCH–L
UC_REX_UNIF
UC_REX_SN
UC_REX_TCAUCH–S
UC_REX_TCAUCH–L
2000
1500
1000
500
t_n186
AC_RAM_UNIF
AC_RAM_SN
AC_RAM_TCAUCH–S
AC_RAM_TCAUCH–L
AC_REX_UNIF
AC_REX_SN
AC_REX_TCAUCH–S
AC_REX_TCAUCH–L
UC_RAM_UNIF
UC_RAM_SN
UC_RAM_TCAUCH–S
UC_RAM_TCAUCH–L
UC_REX_UNIF
UC_REX_SN
UC_REX_TCAUCH–S
UC_REX_TCAUCH–L
600
400
200
t_n190
AC_RAM_UNIF
AC_RAM_SN
AC_RAM_TCAUCH–S
AC_RAM_TCAUCH–L
AC_REX_UNIF
AC_REX_SN
AC_REX_TCAUCH–S
AC_REX_TCAUCH–L
UC_RAM_UNIF
UC_RAM_SN
UC_RAM_TCAUCH–S
UC_RAM_TCAUCH–L
UC_REX_UNIF
UC_REX_SN
UC_REX_TCAUCH–S
UC_REX_TCAUCH–L
3500
3000
2500
2000
t_n187
AC_RAM_UNIF
AC_RAM_SN
AC_RAM_TCAUCH–S
AC_RAM_TCAUCH–L
AC_REX_UNIF
AC_REX_SN
AC_REX_TCAUCH–S
AC_REX_TCAUCH–L
UC_RAM_UNIF
UC_RAM_SN
UC_RAM_TCAUCH–S
UC_RAM_TCAUCH–L
UC_REX_UNIF
UC_REX_SN
UC_REX_TCAUCH–S
UC_REX_TCAUCH–L
2500
2000
1500
1000
500
t_n191
AC_RAM_UNIF
AC_RAM_SN
AC_RAM_TCAUCH–S
AC_RAM_TCAUCH–L
AC_REX_UNIF
AC_REX_SN
AC_REX_TCAUCH–S
AC_REX_TCAUCH–L
UC_RAM_UNIF
UC_RAM_SN
UC_RAM_TCAUCH–S
UC_RAM_TCAUCH–L
UC_REX_UNIF
UC_REX_SN
UC_REX_TCAUCH–S
UC_REX_TCAUCH–L
3000
2500
2000
t_n188
AC_RAM_UNIF
AC_RAM_SN
AC_RAM_TCAUCH–S
AC_RAM_TCAUCH–L
AC_REX_UNIF
AC_REX_SN
AC_REX_TCAUCH–S
AC_REX_TCAUCH–L
UC_RAM_UNIF
UC_RAM_SN
UC_RAM_TCAUCH–S
UC_RAM_TCAUCH–L
UC_REX_UNIF
UC_REX_SN
UC_REX_TCAUCH–S
UC_REX_TCAUCH–L
2000
1500
1000
500
t_n192
AC_RAM_UNIF
AC_RAM_SN
AC_RAM_TCAUCH–S
AC_RAM_TCAUCH–L
AC_REX_UNIF
AC_REX_SN
AC_REX_TCAUCH–S
AC_REX_TCAUCH–L
UC_RAM_UNIF
UC_RAM_SN
UC_RAM_TCAUCH–S
UC_RAM_TCAUCH–L
UC_REX_UNIF
UC_REX_SN
UC_REX_TCAUCH–S
UC_REX_TCAUCH–L
3000
2500
2000
1500

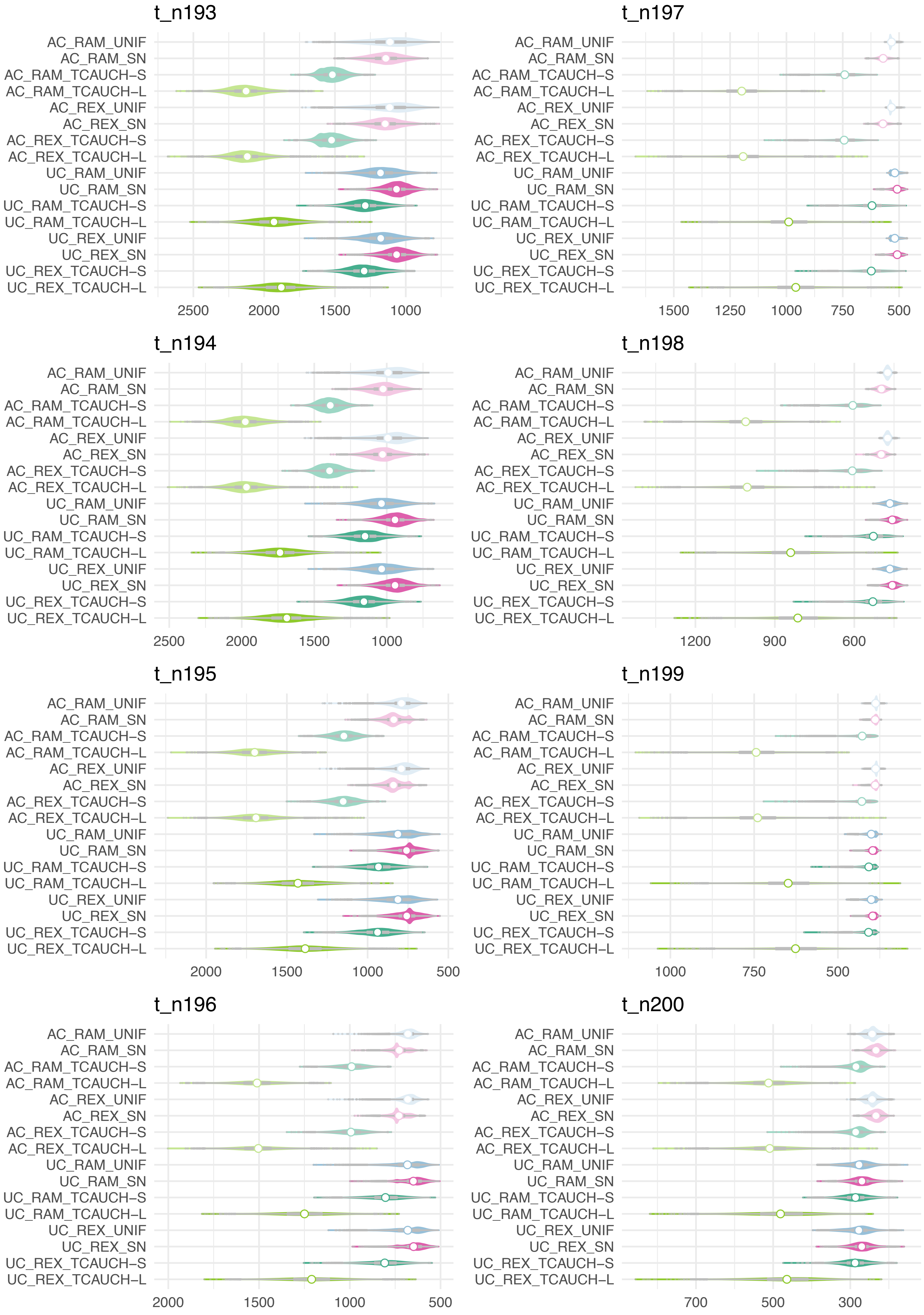

t_n193
t_n197
t_n194
t_n198
t_n195
t_n199
t_n196
t_n200
AC_RAM_UNIF
AC_RAM_SN
AC_RAM_TCAUCH−S
AC_RAM_TCAUCH−L
AC_REX_UNIF
AC_REX_SN
AC_REX_TCAUCH−S
AC_REX_TCAUCH−L
UC_RAM_UNIF
UC_RAM_SN
UC_RAM_TCAUCH−S
UC_RAM_TCAUCH−L
UC_REX_UNIF
UC_REX_SN
UC_REX_TCAUCH−S
UC_REX_TCAUCH−L
2500 2000 1500 1000
1500 1250 1000 750 500
2500 2000 1500 1000
1200 900 600
2000 1500 1000 500
1000 750 500
2000 1500 1000 500
700 500 300

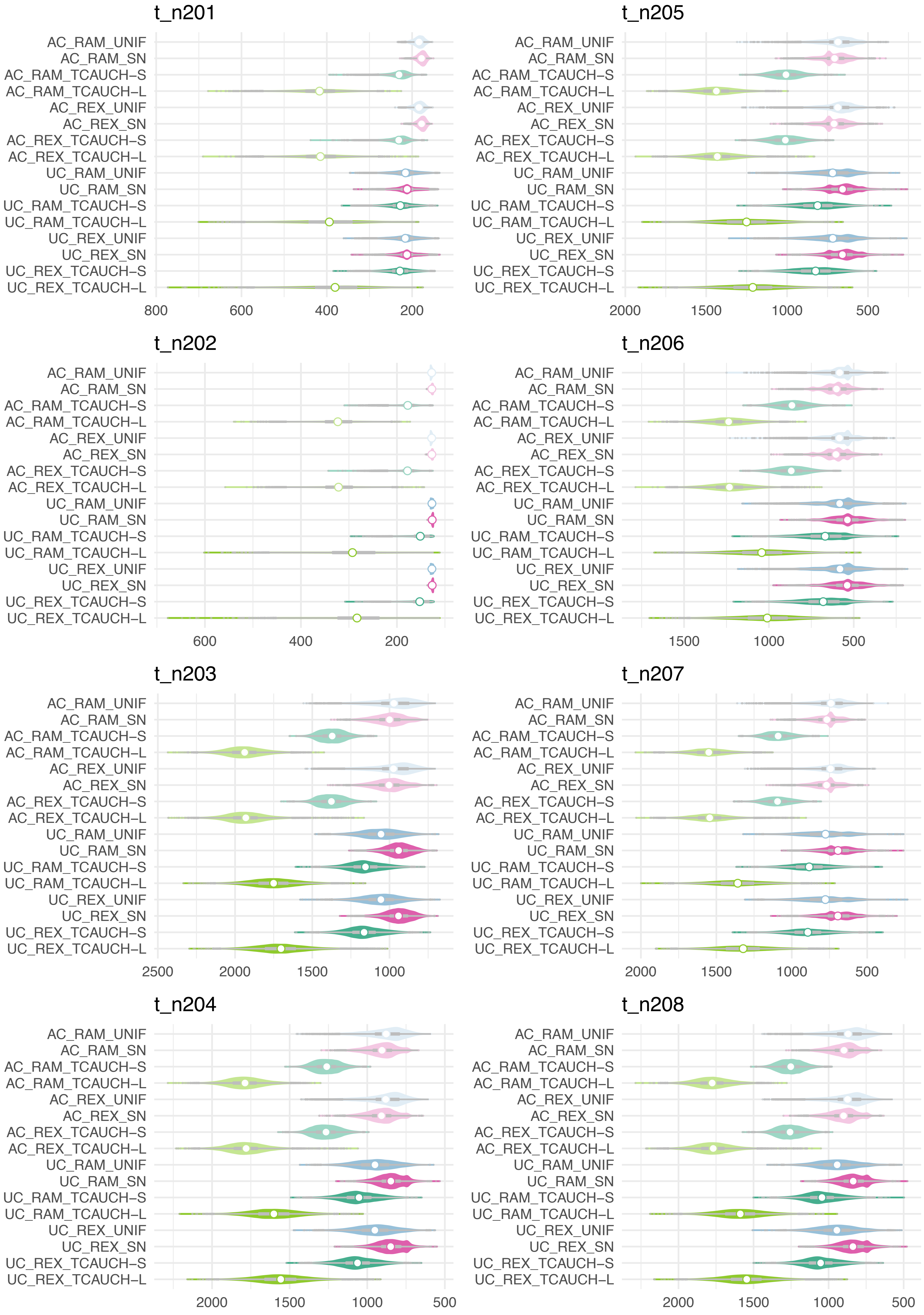
t_n201
AC_RAM_UNIF
AC_RAM_SN
AC_RAM_TCAUCH–S
AC_RAM_TCAUCH–L
AC_REX_UNIF
AC_REX_SN
AC_REX_TCAUCH–S
AC_REX_TCAUCH–L
UC_RAM_UNIF
UC_RAM_SN
UC_RAM_TCAUCH–S
UC_RAM_TCAUCH–L
UC_REX_UNIF
UC_REX_SN
UC_REX_TCAUCH–S
UC_REX_TCAUCH–L
800
600
400
200
t_n205
AC_RAM_UNIF
AC_RAM_SN
AC_RAM_TCAUCH–S
AC_RAM_TCAUCH–L
AC_REX_UNIF
AC_REX_SN
AC_REX_TCAUCH–S
AC_REX_TCAUCH–L
UC_RAM_UNIF
UC_RAM_SN
UC_RAM_TCAUCH–S
UC_RAM_TCAUCH–L
UC_REX_UNIF
UC_REX_SN
UC_REX_TCAUCH–S
UC_REX_TCAUCH–L
2000
1500
1000
500
t_n202
AC_RAM_UNIF
AC_RAM_SN
AC_RAM_TCAUCH–S
AC_RAM_TCAUCH–L
AC_REX_UNIF
AC_REX_SN
AC_REX_TCAUCH–S
AC_REX_TCAUCH–L
UC_RAM_UNIF
UC_RAM_SN
UC_RAM_TCAUCH–S
UC_RAM_TCAUCH–L
UC_REX_UNIF
UC_REX_SN
UC_REX_TCAUCH–S
UC_REX_TCAUCH–L
600
400
200
t_n206
AC_RAM_UNIF
AC_RAM_SN
AC_RAM_TCAUCH–S
AC_RAM_TCAUCH–L
AC_REX_UNIF
AC_REX_SN
AC_REX_TCAUCH–S
AC_REX_TCAUCH–L
UC_RAM_UNIF
UC_RAM_SN
UC_RAM_TCAUCH–S
UC_RAM_TCAUCH–L
UC_REX_UNIF
UC_REX_SN
UC_REX_TCAUCH–S
UC_REX_TCAUCH–L
1500
1000
500
t_n203
AC_RAM_UNIF
AC_RAM_SN
AC_RAM_TCAUCH–S
AC_RAM_TCAUCH–L
AC_REX_UNIF
AC_REX_SN
AC_REX_TCAUCH–S
AC_REX_TCAUCH–L
UC_RAM_UNIF
UC_RAM_SN
UC_RAM_TCAUCH–S
UC_RAM_TCAUCH–L
UC_REX_UNIF
UC_REX_SN
UC_REX_TCAUCH–S
UC_REX_TCAUCH–L
2500
2000
1500
1000
t_n207
AC_RAM_UNIF
AC_RAM_SN
AC_RAM_TCAUCH–S
AC_RAM_TCAUCH–L
AC_REX_UNIF
AC_REX_SN
AC_REX_TCAUCH–S
AC_REX_TCAUCH–L
UC_RAM_UNIF
UC_RAM_SN
UC_RAM_TCAUCH–S
UC_RAM_TCAUCH–L
UC_REX_UNIF
UC_REX_SN
UC_REX_TCAUCH–S
UC_REX_TCAUCH–L
2000
1500
1000
500
t_n204
AC_RAM_UNIF
AC_RAM_SN
AC_RAM_TCAUCH–S
AC_RAM_TCAUCH–L
AC_REX_UNIF
AC_REX_SN
AC_REX_TCAUCH–S
AC_REX_TCAUCH–L
UC_RAM_UNIF
UC_RAM_SN
UC_RAM_TCAUCH–S
UC_RAM_TCAUCH–L
UC_REX_UNIF
UC_REX_SN
UC_REX_TCAUCH–S
UC_REX_TCAUCH–L
2000
1500
1000
500
t_n208
AC_RAM_UNIF
AC_RAM_SN
AC_RAM_TCAUCH–S
AC_RAM_TCAUCH–L
AC_REX_UNIF
AC_REX_SN
AC_REX_TCAUCH–S
AC_REX_TCAUCH–L
UC_RAM_UNIF
UC_RAM_SN
UC_RAM_TCAUCH–S
UC_RAM_TCAUCH–L
UC_REX_UNIF
UC_REX_SN
UC_REX_TCAUCH–S
UC_REX_TCAUCH–L
2000
1500
1000
500

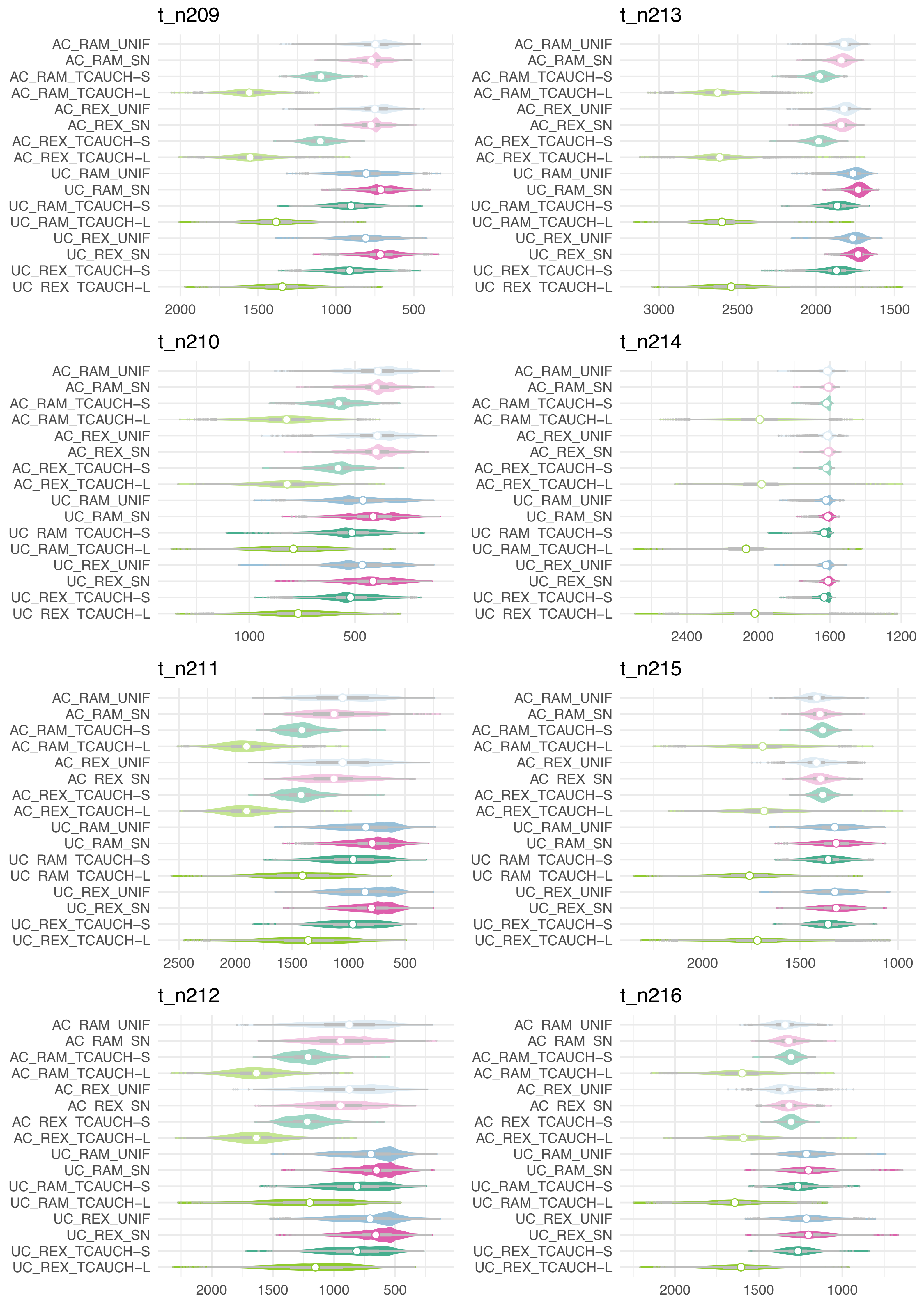
t_n209
AC_RAM_UNIF
AC_RAM_SN
AC_RAM_TCAUCH–S
AC_RAM_TCAUCH–L
AC_REX_UNIF
AC_REX_SN
AC_REX_TCAUCH–S
AC_REX_TCAUCH–L
UC_RAM_UNIF
UC_RAM_SN
UC_RAM_TCAUCH–S
UC_RAM_TCAUCH–L
UC_REX_UNIF
UC_REX_SN
UC_REX_TCAUCH–S
UC_REX_TCAUCH–L
2000
1500
1000
500
t_n213
AC_RAM_UNIF
AC_RAM_SN
AC_RAM_TCAUCH–S
AC_RAM_TCAUCH–L
AC_REX_UNIF
AC_REX_SN
AC_REX_TCAUCH–S
AC_REX_TCAUCH–L
UC_RAM_UNIF
UC_RAM_SN
UC_RAM_TCAUCH–S
UC_RAM_TCAUCH–L
UC_REX_UNIF
UC_REX_SN
UC_REX_TCAUCH–S
UC_REX_TCAUCH–L
3000
2500
2000
1500
t_n210
AC_RAM_UNIF
AC_RAM_SN
AC_RAM_TCAUCH–S
AC_RAM_TCAUCH–L
AC_REX_UNIF
AC_REX_SN
AC_REX_TCAUCH–S
AC_REX_TCAUCH–L
UC_RAM_UNIF
UC_RAM_SN
UC_RAM_TCAUCH–S
UC_RAM_TCAUCH–L
UC_REX_UNIF
UC_REX_SN
UC_REX_TCAUCH–S
UC_REX_TCAUCH–L
1000
500
t_n214
AC_RAM_UNIF
AC_RAM_SN
AC_RAM_TCAUCH–S
AC_RAM_TCAUCH–L
AC_REX_UNIF
AC_REX_SN
AC_REX_TCAUCH–S
AC_REX_TCAUCH–L
UC_RAM_UNIF
UC_RAM_SN
UC_RAM_TCAUCH–S
UC_RAM_TCAUCH–L
UC_REX_UNIF
UC_REX_SN
UC_REX_TCAUCH–S
UC_REX_TCAUCH–L
2400
2000
1600
1200
t_n211
AC_RAM_UNIF
AC_RAM_SN
AC_RAM_TCAUCH–S
AC_RAM_TCAUCH–L
AC_REX_UNIF
AC_REX_SN
AC_REX_TCAUCH–S
AC_REX_TCAUCH–L
UC_RAM_UNIF
UC_RAM_SN
UC_RAM_TCAUCH–S
UC_RAM_TCAUCH–L
UC_REX_UNIF
UC_REX_SN
UC_REX_TCAUCH–S
UC_REX_TCAUCH–L
2500
2000
1500
1000
500
t_n215
AC_RAM_UNIF
AC_RAM_SN
AC_RAM_TCAUCH–S
AC_RAM_TCAUCH–L
AC_REX_UNIF
AC_REX_SN
AC_REX_TCAUCH–S
AC_REX_TCAUCH–L
UC_RAM_UNIF
UC_RAM_SN
UC_RAM_TCAUCH–S
UC_RAM_TCAUCH–L
UC_REX_UNIF
UC_REX_SN
UC_REX_TCAUCH–S
UC_REX_TCAUCH–L
2000
1500
1000
t_n212
AC_RAM_UNIF
AC_RAM_SN
AC_RAM_TCAUCH–S
AC_RAM_TCAUCH–L
AC_REX_UNIF
AC_REX_SN
AC_REX_TCAUCH–S
AC_REX_TCAUCH–L
UC_RAM_UNIF
UC_RAM_SN
UC_RAM_TCAUCH–S
UC_RAM_TCAUCH–L
UC_REX_UNIF
UC_REX_SN
UC_REX_TCAUCH–S
UC_REX_TCAUCH–L
2000
1500
1000
500
t_n216
AC_RAM_UNIF
AC_RAM_SN
AC_RAM_TCAUCH–S
AC_RAM_TCAUCH–L
AC_REX_UNIF
AC_REX_SN
AC_REX_TCAUCH–S
AC_REX_TCAUCH–L
UC_RAM_UNIF
UC_RAM_SN
UC_RAM_TCAUCH–S
UC_RAM_TCAUCH–L
UC_REX_UNIF
UC_REX_SN
UC_REX_TCAUCH–S
UC_REX_TCAUCH–L
2000
1500
1000

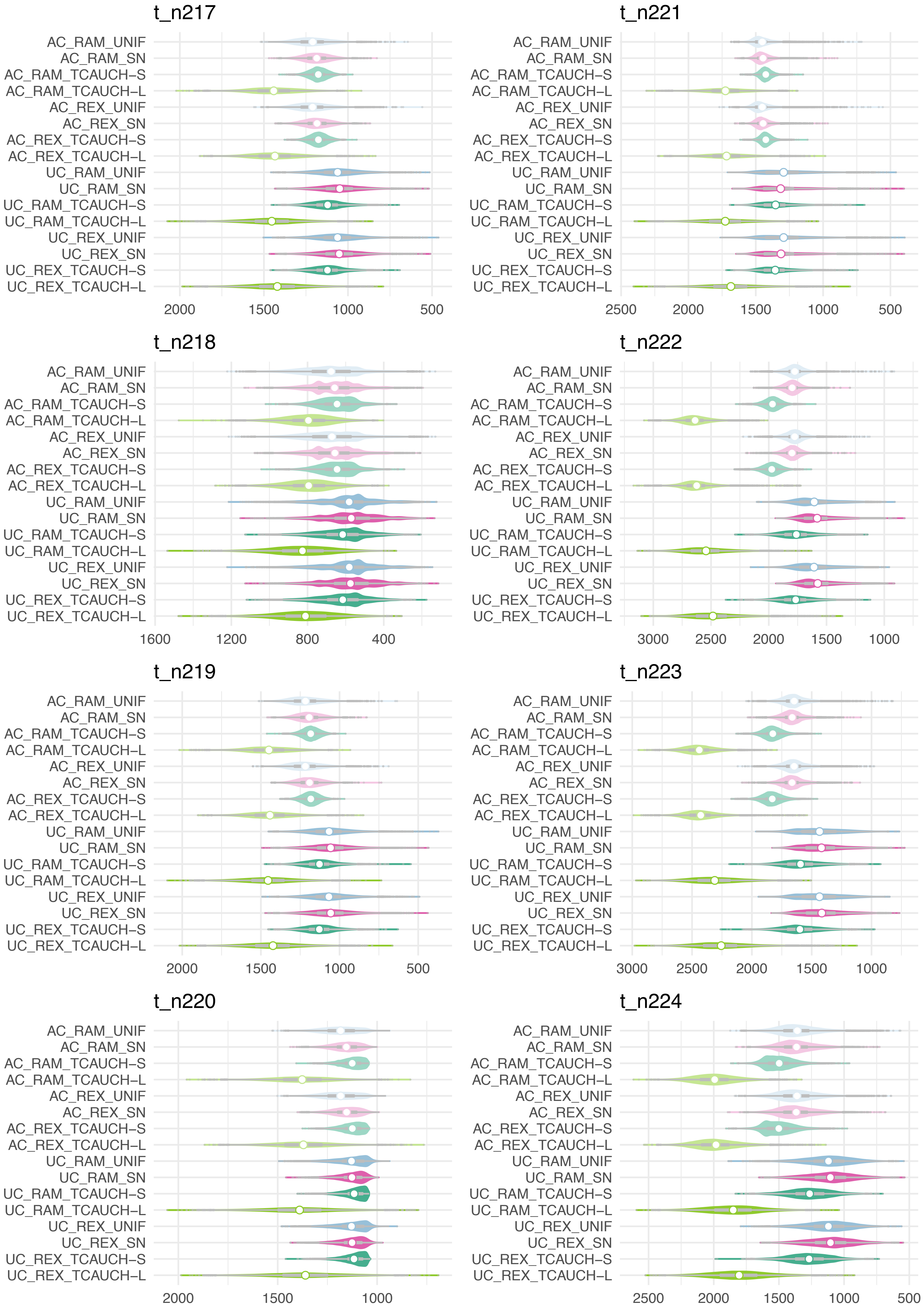

t_n217
AC_RAM_UNIF
AC_RAM_SN
AC_RAM_TCAUCH−S
AC_RAM_TCAUCH−L
AC_REX_UNIF
AC_REX_SN
AC_REX_TCAUCH−S
AC_REX_TCAUCH−L
UC_RAM_UNIF
UC_RAM_SN
UC_RAM_TCAUCH−S
UC_RAM_TCAUCH−L
UC_REX_UNIF
UC_REX_SN
UC_REX_TCAUCH−S
UC_REX_TCAUCH−L
2000
1500
1000
500
t_n221
AC_RAM_UNIF
AC_RAM_SN
AC_RAM_TCAUCH−S
AC_RAM_TCAUCH−L
AC_REX_UNIF
AC_REX_SN
AC_REX_TCAUCH−S
AC_REX_TCAUCH−L
UC_RAM_UNIF
UC_RAM_SN
UC_RAM_TCAUCH−S
UC_RAM_TCAUCH−L
UC_REX_UNIF
UC_REX_SN
UC_REX_TCAUCH−S
UC_REX_TCAUCH−L
2500
2000
1500
1000
500
t_n218
AC_RAM_UNIF
AC_RAM_SN
AC_RAM_TCAUCH−S
AC_RAM_TCAUCH−L
AC_REX_UNIF
AC_REX_SN
AC_REX_TCAUCH−S
AC_REX_TCAUCH−L
UC_RAM_UNIF
UC_RAM_SN
UC_RAM_TCAUCH−S
UC_RAM_TCAUCH−L
UC_REX_UNIF
UC_REX_SN
UC_REX_TCAUCH−S
UC_REX_TCAUCH−L
1600
1200
800
400
t_n222
AC_RAM_UNIF
AC_RAM_SN
AC_RAM_TCAUCH−S
AC_RAM_TCAUCH−L
AC_REX_UNIF
AC_REX_SN
AC_REX_TCAUCH−S
AC_REX_TCAUCH−L
UC_RAM_UNIF
UC_RAM_SN
UC_RAM_TCAUCH−S
UC_RAM_TCAUCH−L
UC_REX_UNIF
UC_REX_SN
UC_REX_TCAUCH−S
UC_REX_TCAUCH−L
3000
2500
2000
1500
1000
t_n219
AC_RAM_UNIF
AC_RAM_SN
AC_RAM_TCAUCH−S
AC_RAM_TCAUCH−L
AC_REX_UNIF
AC_REX_SN
AC_REX_TCAUCH−S
AC_REX_TCAUCH−L
UC_RAM_UNIF
UC_RAM_SN
UC_RAM_TCAUCH−S
UC_RAM_TCAUCH−L
UC_REX_UNIF
UC_REX_SN
UC_REX_TCAUCH−S
UC_REX_TCAUCH−L
2000
1500
1000
500
t_n223
AC_RAM_UNIF
AC_RAM_SN
AC_RAM_TCAUCH−S
AC_RAM_TCAUCH−L
AC_REX_UNIF
AC_REX_SN
AC_REX_TCAUCH−S
AC_REX_TCAUCH−L
UC_RAM_UNIF
UC_RAM_SN
UC_RAM_TCAUCH−S
UC_RAM_TCAUCH−L
UC_REX_UNIF
UC_REX_SN
UC_REX_TCAUCH−S
UC_REX_TCAUCH−L
3000
2500
2000
1500
1000
t_n220
AC_RAM_UNIF
AC_RAM_SN
AC_RAM_TCAUCH−S
AC_RAM_TCAUCH−L
AC_REX_UNIF
AC_REX_SN
AC_REX_TCAUCH−S
AC_REX_TCAUCH−L
UC_RAM_UNIF
UC_RAM_SN
UC_RAM_TCAUCH−S
UC_RAM_TCAUCH−L
UC_REX_UNIF
UC_REX_SN
UC_REX_TCAUCH−S
UC_REX_TCAUCH−L
2000
1500
1000
t_n224
AC_RAM_UNIF
AC_RAM_SN
AC_RAM_TCAUCH−S
AC_RAM_TCAUCH−L
AC_REX_UNIF
AC_REX_SN
AC_REX_TCAUCH−S
AC_REX_TCAUCH−L
UC_RAM_UNIF
UC_RAM_SN
UC_RAM_TCAUCH−S
UC_RAM_TCAUCH−L
UC_REX_UNIF
UC_REX_SN
UC_REX_TCAUCH−S
UC_REX_TCAUCH−L
2500
2000
1500
1000
500

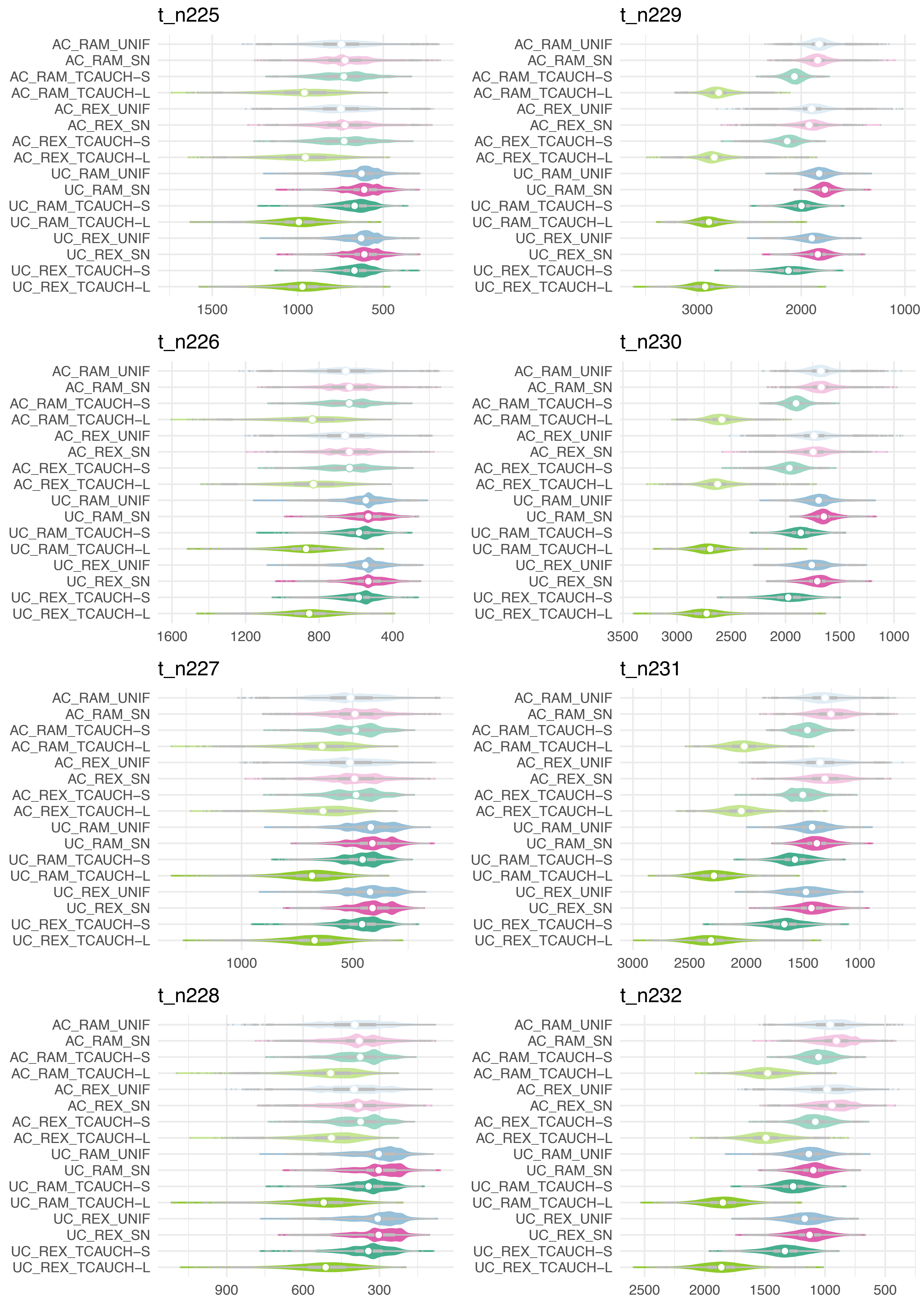
t_n225
AC_RAM_UNIF
AC_RAM_SN
AC_RAM_TCAUCH−S
AC_RAM_TCAUCH−L
AC_REX_UNIF
AC_REX_SN
AC_REX_TCAUCH−S
AC_REX_TCAUCH−L
UC_RAM_UNIF
UC_RAM_SN
UC_RAM_TCAUCH−S
UC_RAM_TCAUCH−L
UC_REX_UNIF
UC_REX_SN
UC_REX_TCAUCH−S
UC_REX_TCAUCH−L
1500
1000
500
t_n229
AC_RAM_UNIF
AC_RAM_SN
AC_RAM_TCAUCH−S
AC_RAM_TCAUCH−L
AC_REX_UNIF
AC_REX_SN
AC_REX_TCAUCH−S
AC_REX_TCAUCH−L
UC_RAM_UNIF
UC_RAM_SN
UC_RAM_TCAUCH−S
UC_RAM_TCAUCH−L
UC_REX_UNIF
UC_REX_SN
UC_REX_TCAUCH−S
UC_REX_TCAUCH−L
3000
2000
1000
t_n226
AC_RAM_UNIF
AC_RAM_SN
AC_RAM_TCAUCH−S
AC_RAM_TCAUCH−L
AC_REX_UNIF
AC_REX_SN
AC_REX_TCAUCH−S
AC_REX_TCAUCH−L
UC_RAM_UNIF
UC_RAM_SN
UC_RAM_TCAUCH−S
UC_RAM_TCAUCH−L
UC_REX_UNIF
UC_REX_SN
UC_REX_TCAUCH−S
UC_REX_TCAUCH−L
1600
1200
800
400
t_n230
AC_RAM_UNIF
AC_RAM_SN
AC_RAM_TCAUCH−S
AC_RAM_TCAUCH−L
AC_REX_UNIF
AC_REX_SN
AC_REX_TCAUCH−S
AC_REX_TCAUCH−L
UC_RAM_UNIF
UC_RAM_SN
UC_RAM_TCAUCH−S
UC_RAM_TCAUCH−L
UC_REX_UNIF
UC_REX_SN
UC_REX_TCAUCH−S
UC_REX_TCAUCH−L
3500
3000
2500
2000
1500
1000
t_n227
AC_RAM_UNIF
AC_RAM_SN
AC_RAM_TCAUCH−S
AC_RAM_TCAUCH−L
AC_REX_UNIF
AC_REX_SN
AC_REX_TCAUCH−S
AC_REX_TCAUCH−L
UC_RAM_UNIF
UC_RAM_SN
UC_RAM_TCAUCH−S
UC_RAM_TCAUCH−L
UC_REX_UNIF
UC_REX_SN
UC_REX_TCAUCH−S
UC_REX_TCAUCH−L
1000
500
t_n231
AC_RAM_UNIF
AC_RAM_SN
AC_RAM_TCAUCH−S
AC_RAM_TCAUCH−L
AC_REX_UNIF
AC_REX_SN
AC_REX_TCAUCH−S
AC_REX_TCAUCH−L
UC_RAM_UNIF
UC_RAM_SN
UC_RAM_TCAUCH−S
UC_RAM_TCAUCH−L
UC_REX_UNIF
UC_REX_SN
UC_REX_TCAUCH−S
UC_REX_TCAUCH−L
3000
2500
2000
1500
1000
t_n228
AC_RAM_UNIF
AC_RAM_SN
AC_RAM_TCAUCH−S
AC_RAM_TCAUCH−L
AC_REX_UNIF
AC_REX_SN
AC_REX_TCAUCH−S
AC_REX_TCAUCH−L
UC_RAM_UNIF
UC_RAM_SN
UC_RAM_TCAUCH−S
UC_RAM_TCAUCH−L
UC_REX_UNIF
UC_REX_SN
UC_REX_TCAUCH−S
UC_REX_TCAUCH−L
900
600
300
t_n232
AC_RAM_UNIF
AC_RAM_SN
AC_RAM_TCAUCH−S
AC_RAM_TCAUCH−L
AC_REX_UNIF
AC_REX_SN
AC_REX_TCAUCH−S
AC_REX_TCAUCH−L
UC_RAM_UNIF
UC_RAM_SN
UC_RAM_TCAUCH−S
UC_RAM_TCAUCH−L
UC_REX_UNIF
UC_REX_SN
UC_REX_TCAUCH−S
UC_REX_TCAUCH−L
2500
2000
1500
1000
500

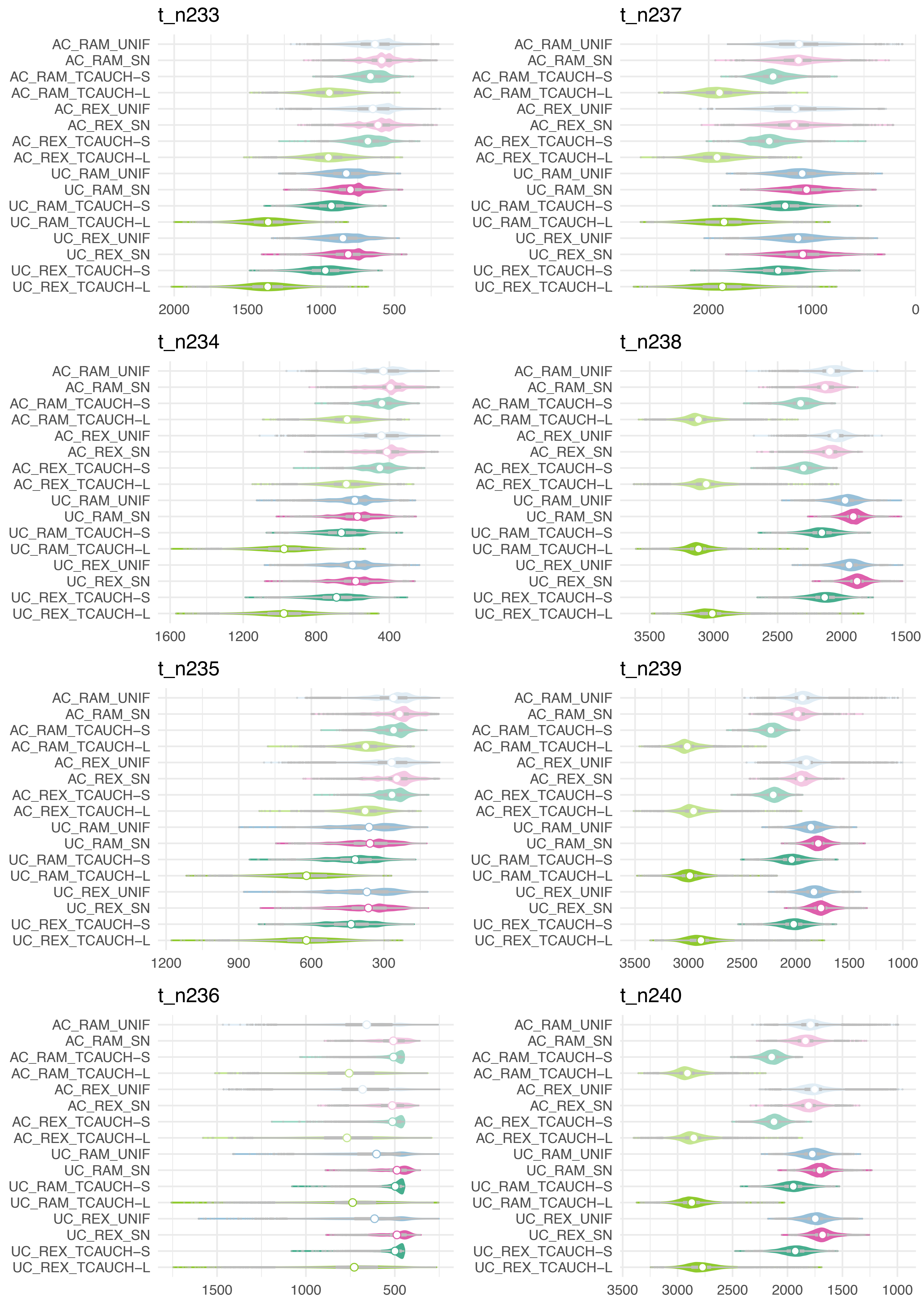

t_n233
AC_RAM_UNIF
AC_RAM_SN
AC_RAM_TCAUCH–S
AC_RAM_TCAUCH–L
AC_REX_UNIF
AC_REX_SN
AC_REX_TCAUCH–S
AC_REX_TCAUCH–L
UC_RAM_UNIF
UC_RAM_SN
UC_RAM_TCAUCH–S
UC_RAM_TCAUCH–L
UC_REX_UNIF
UC_REX_SN
UC_REX_TCAUCH–S
UC_REX_TCAUCH–L
2000
1500
1000
500
t_n237
AC_RAM_UNIF
AC_RAM_SN
AC_RAM_TCAUCH–S
AC_RAM_TCAUCH–L
AC_REX_UNIF
AC_REX_SN
AC_REX_TCAUCH–S
AC_REX_TCAUCH–L
UC_RAM_UNIF
UC_RAM_SN
UC_RAM_TCAUCH–S
UC_RAM_TCAUCH–L
UC_REX_UNIF
UC_REX_SN
UC_REX_TCAUCH–S
UC_REX_TCAUCH–L
2000
1000
0
t_n234
AC_RAM_UNIF
AC_RAM_SN
AC_RAM_TCAUCH–S
AC_RAM_TCAUCH–L
AC_REX_UNIF
AC_REX_SN
AC_REX_TCAUCH–S
AC_REX_TCAUCH–L
UC_RAM_UNIF
UC_RAM_SN
UC_RAM_TCAUCH–S
UC_RAM_TCAUCH–L
UC_REX_UNIF
UC_REX_SN
UC_REX_TCAUCH–S
UC_REX_TCAUCH–L
1600
1200
800
400
t_n238
AC_RAM_UNIF
AC_RAM_SN
AC_RAM_TCAUCH–S
AC_RAM_TCAUCH–L
AC_REX_UNIF
AC_REX_SN
AC_REX_TCAUCH–S
AC_REX_TCAUCH–L
UC_RAM_UNIF
UC_RAM_SN
UC_RAM_TCAUCH–S
UC_RAM_TCAUCH–L
UC_REX_UNIF
UC_REX_SN
UC_REX_TCAUCH–S
UC_REX_TCAUCH–L
3500
3000
2500
2000
1500
t_n235
AC_RAM_UNIF
AC_RAM_SN
AC_RAM_TCAUCH–S
AC_RAM_TCAUCH–L
AC_REX_UNIF
AC_REX_SN
AC_REX_TCAUCH–S
AC_REX_TCAUCH–L
UC_RAM_UNIF
UC_RAM_SN
UC_RAM_TCAUCH–S
UC_RAM_TCAUCH–L
UC_REX_UNIF
UC_REX_SN
UC_REX_TCAUCH–S
UC_REX_TCAUCH–L
1200
900
600
300
t_n239
AC_RAM_UNIF
AC_RAM_SN
AC_RAM_TCAUCH–S
AC_RAM_TCAUCH–L
AC_REX_UNIF
AC_REX_SN
AC_REX_TCAUCH–S
AC_REX_TCAUCH–L
UC_RAM_UNIF
UC_RAM_SN
UC_RAM_TCAUCH–S
UC_RAM_TCAUCH–L
UC_REX_UNIF
UC_REX_SN
UC_REX_TCAUCH–S
UC_REX_TCAUCH–L
3500
3000
2500
2000
1500
1000
t_n236
AC_RAM_UNIF
AC_RAM_SN
AC_RAM_TCAUCH–S
AC_RAM_TCAUCH–L
AC_REX_UNIF
AC_REX_SN
AC_REX_TCAUCH–S
AC_REX_TCAUCH–L
UC_RAM_UNIF
UC_RAM_SN
UC_RAM_TCAUCH–S
UC_RAM_TCAUCH–L
UC_REX_UNIF
UC_REX_SN
UC_REX_TCAUCH–S
UC_REX_TCAUCH–L
1500
1000
500
t_n240
AC_RAM_UNIF
AC_RAM_SN
AC_RAM_TCAUCH–S
AC_RAM_TCAUCH–L
AC_REX_UNIF
AC_REX_SN
AC_REX_TCAUCH–S
AC_REX_TCAUCH–L
UC_RAM_UNIF
UC_RAM_SN
UC_RAM_TCAUCH–S
UC_RAM_TCAUCH–L
UC_REX_UNIF
UC_REX_SN
UC_REX_TCAUCH–S
UC_REX_TCAUCH–L
3500
3000
2500
2000
1500
1000

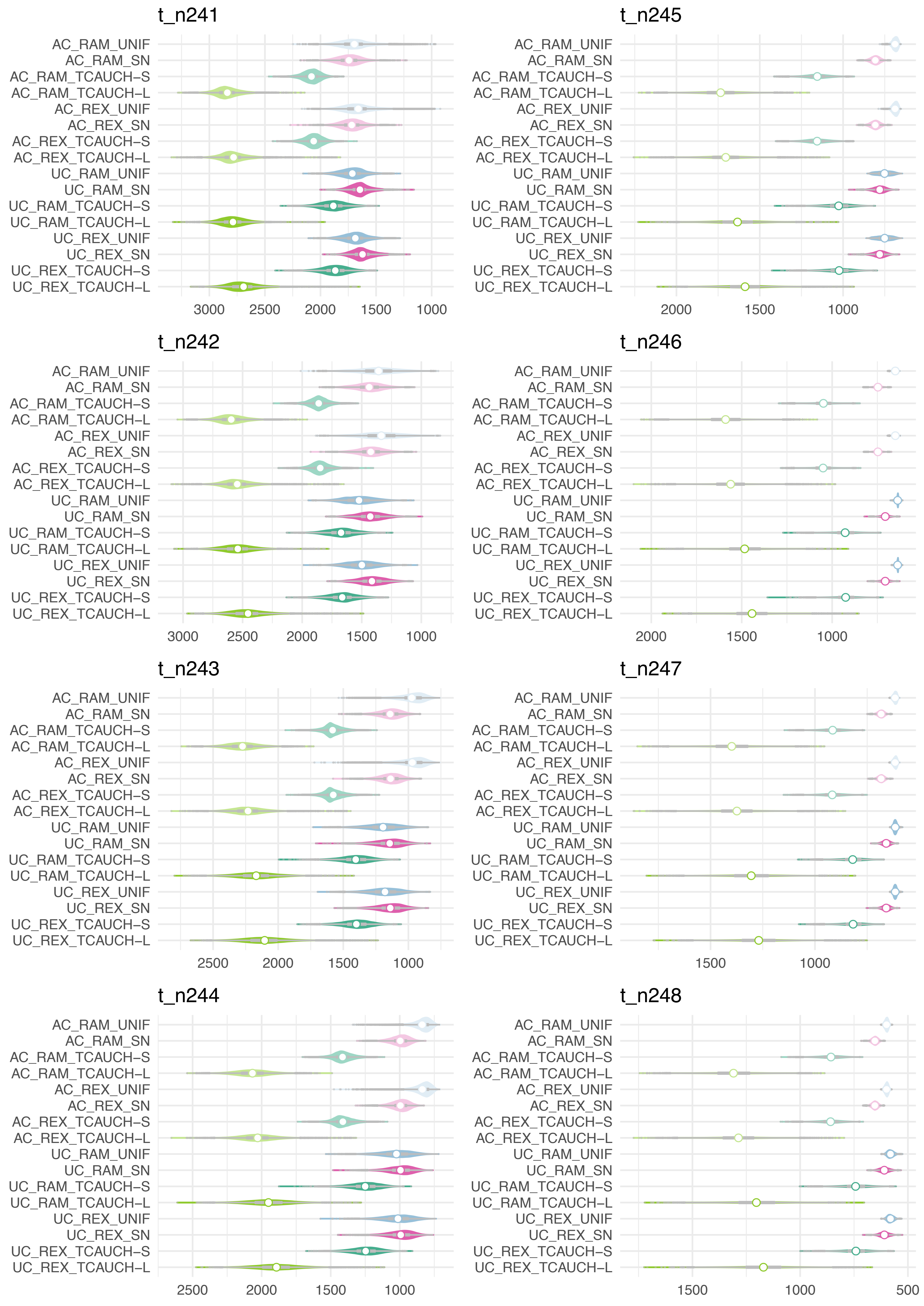
t_n241
AC_RAM_UNIF
AC_RAM_SN
AC_RAM_TCAUCH–S
AC_RAM_TCAUCH–L
AC_REX_UNIF
AC_REX_SN
AC_REX_TCAUCH–S
AC_REX_TCAUCH–L
UC_RAM_UNIF
UC_RAM_SN
UC_RAM_TCAUCH–S
UC_RAM_TCAUCH–L
UC_REX_UNIF
UC_REX_SN
UC_REX_TCAUCH–S
UC_REX_TCAUCH–L
3000 2500 2000 1500 1000
t_n245
2000 1500 1000
t_n242
3000 2500 2000 1500 1000
t_n246
2000 1500 1000
t_n243
2500 2000 1500 1000
t_n247
1500 1000
t_n244
2500 2000 1500 1000
t_n248
1500 1000 500

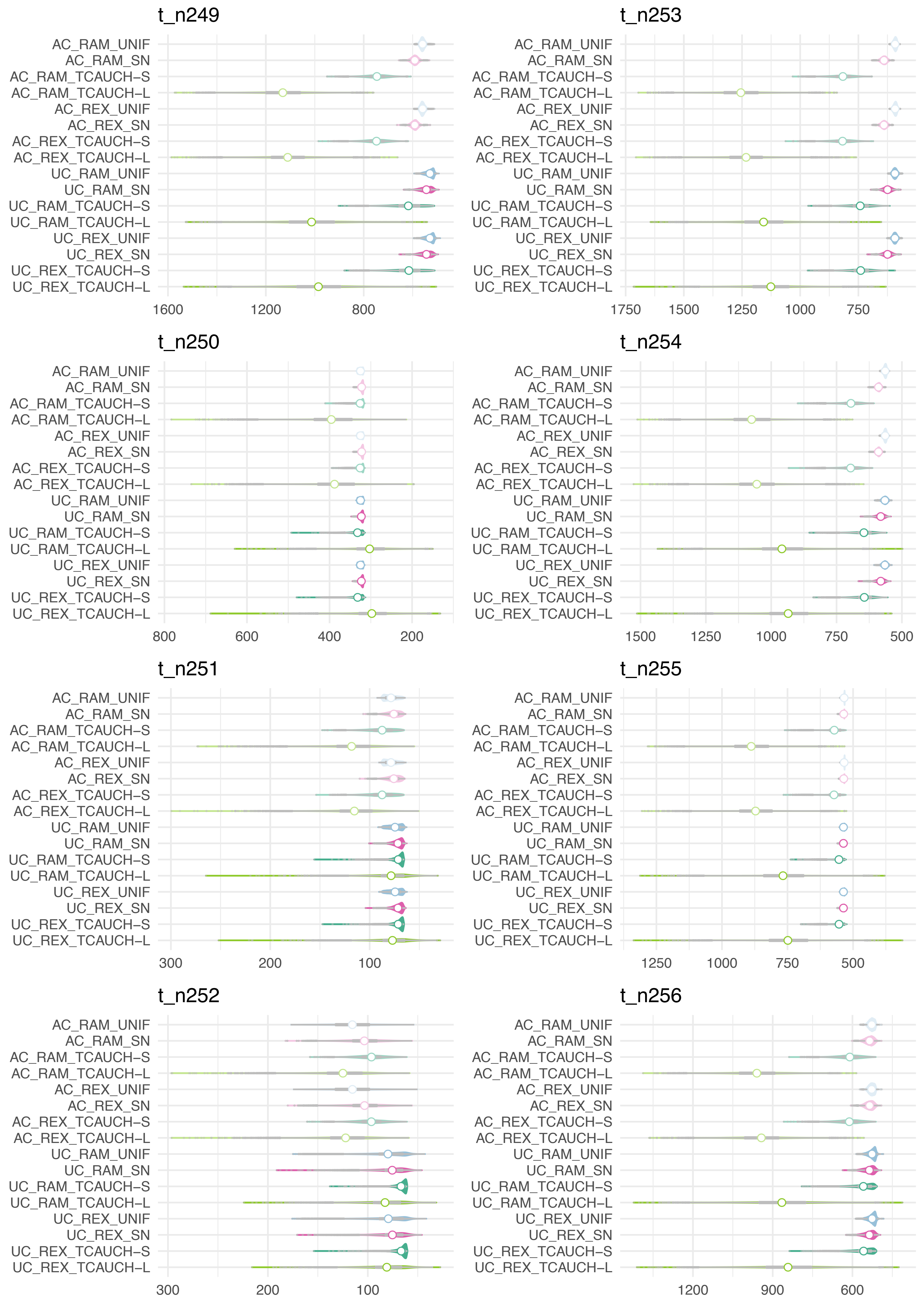
t_n249
AC_RAM_UNIF
AC_RAM_SN
AC_RAM_TCAUCH−S
AC_RAM_TCAUCH−L
AC_REX_UNIF
AC_REX_SN
AC_REX_TCAUCH−S
AC_REX_TCAUCH−L
UC_RAM_UNIF
UC_RAM_SN
UC_RAM_TCAUCH−S
UC_RAM_TCAUCH−L
UC_REX_UNIF
UC_REX_SN
UC_REX_TCAUCH−S
UC_REX_TCAUCH−L
1600
1200
800
t_n253
AC_RAM_UNIF
AC_RAM_SN
AC_RAM_TCAUCH−S
AC_RAM_TCAUCH−L
AC_REX_UNIF
AC_REX_SN
AC_REX_TCAUCH−S
AC_REX_TCAUCH−L
UC_RAM_UNIF
UC_RAM_SN
UC_RAM_TCAUCH−S
UC_RAM_TCAUCH−L
UC_REX_UNIF
UC_REX_SN
UC_REX_TCAUCH−S
UC_REX_TCAUCH−L
1750
1500
1250
1000
750
t_n250
AC_RAM_UNIF
AC_RAM_SN
AC_RAM_TCAUCH−S
AC_RAM_TCAUCH−L
AC_REX_UNIF
AC_REX_SN
AC_REX_TCAUCH−S
AC_REX_TCAUCH−L
UC_RAM_UNIF
UC_RAM_SN
UC_RAM_TCAUCH−S
UC_RAM_TCAUCH−L
UC_REX_UNIF
UC_REX_SN
UC_REX_TCAUCH−S
UC_REX_TCAUCH−L
800
600
400
200
t_n254
AC_RAM_UNIF
AC_RAM_SN
AC_RAM_TCAUCH−S
AC_RAM_TCAUCH−L
AC_REX_UNIF
AC_REX_SN
AC_REX_TCAUCH−S
AC_REX_TCAUCH−L
UC_RAM_UNIF
UC_RAM_SN
UC_RAM_TCAUCH−S
UC_RAM_TCAUCH−L
UC_REX_UNIF
UC_REX_SN
UC_REX_TCAUCH−S
UC_REX_TCAUCH−L
1500
1250
1000
750
500
t_n251
AC_RAM_UNIF
AC_RAM_SN
AC_RAM_TCAUCH−S
AC_RAM_TCAUCH−L
AC_REX_UNIF
AC_REX_SN
AC_REX_TCAUCH−S
AC_REX_TCAUCH−L
UC_RAM_UNIF
UC_RAM_SN
UC_RAM_TCAUCH−S
UC_RAM_TCAUCH−L
UC_REX_UNIF
UC_REX_SN
UC_REX_TCAUCH−S
UC_REX_TCAUCH−L
300
200
100
t_n255
AC_RAM_UNIF
AC_RAM_SN
AC_RAM_TCAUCH−S
AC_RAM_TCAUCH−L
AC_REX_UNIF
AC_REX_SN
AC_REX_TCAUCH−S
AC_REX_TCAUCH−L
UC_RAM_UNIF
UC_RAM_SN
UC_RAM_TCAUCH−S
UC_RAM_TCAUCH−L
UC_REX_UNIF
UC_REX_SN
UC_REX_TCAUCH−S
UC_REX_TCAUCH−L
1250
1000
750
500
t_n252
AC_RAM_UNIF
AC_RAM_SN
AC_RAM_TCAUCH−S
AC_RAM_TCAUCH−L
AC_REX_UNIF
AC_REX_SN
AC_REX_TCAUCH−S
AC_REX_TCAUCH−L
UC_RAM_UNIF
UC_RAM_SN
UC_RAM_TCAUCH−S
UC_RAM_TCAUCH−L
UC_REX_UNIF
UC_REX_SN
UC_REX_TCAUCH−S
UC_REX_TCAUCH−L
300
200
100
t_n256
AC_RAM_UNIF
AC_RAM_SN
AC_RAM_TCAUCH−S
AC_RAM_TCAUCH−L
AC_REX_UNIF
AC_REX_SN
AC_REX_TCAUCH−S
AC_REX_TCAUCH−L
UC_RAM_UNIF
UC_RAM_SN
UC_RAM_TCAUCH−S
UC_RAM_TCAUCH−L
UC_REX_UNIF
UC_REX_SN
UC_REX_TCAUCH−S
UC_REX_TCAUCH−L
1200
900
600

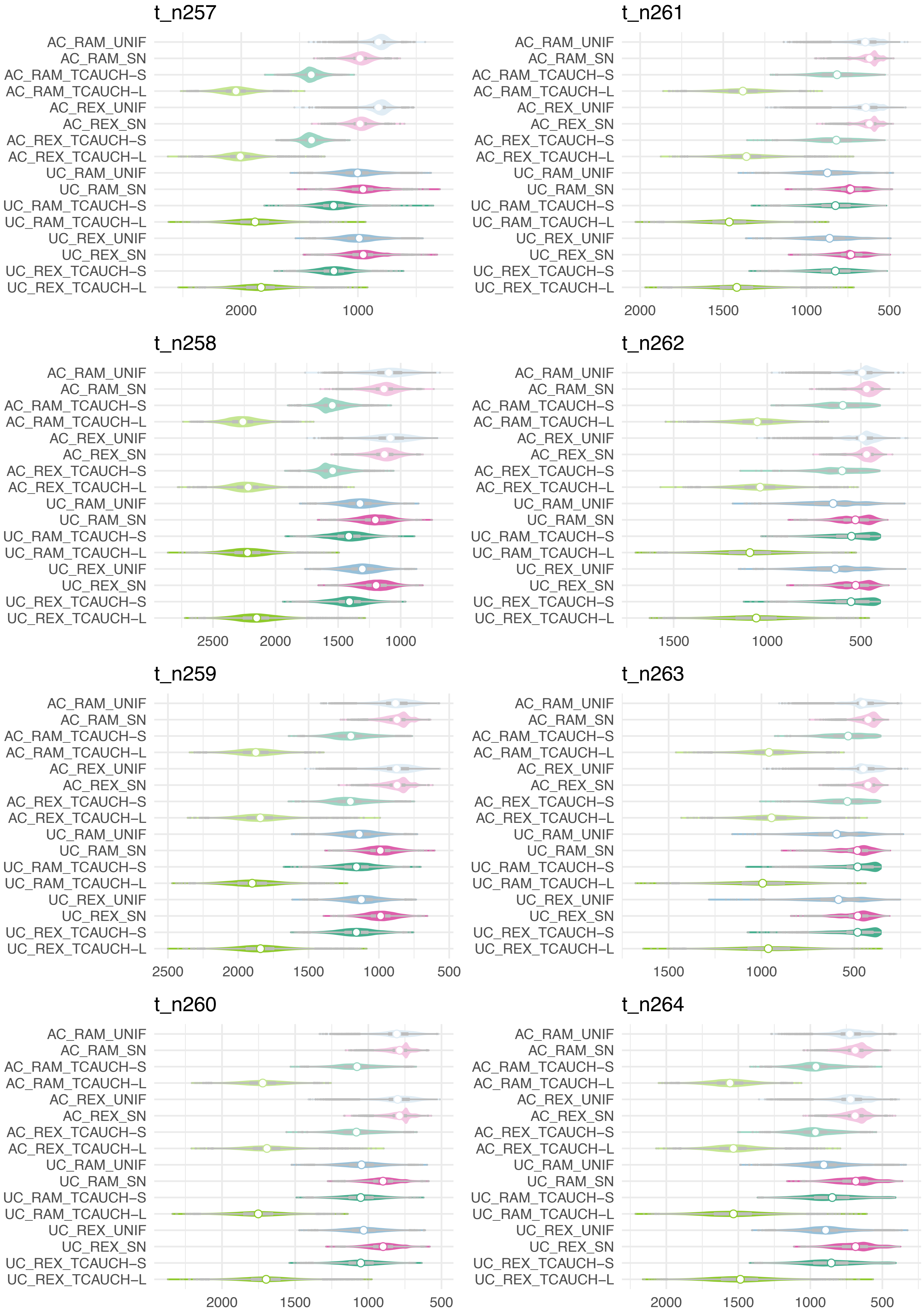
t_n257
t_n261
t_n258
t_n262
t_n259
t_n263
t_n260
t_n264
AC_RAM_UNIF
AC_RAM_SN
AC_RAM_TCAUCH-S
AC_RAM_TCAUCH-L
AC_REX_UNIF
AC_REX_SN
AC_REX_TCAUCH-S
AC_REX_TCAUCH-L
UC_RAM_UNIF
UC_RAM_SN
UC_RAM_TCAUCH-S
UC_RAM_TCAUCH-L
UC_REX_UNIF
UC_REX_SN
UC_REX_TCAUCH-S
UC_REX_TCAUCH-L
2500
2000
1500
1000
500

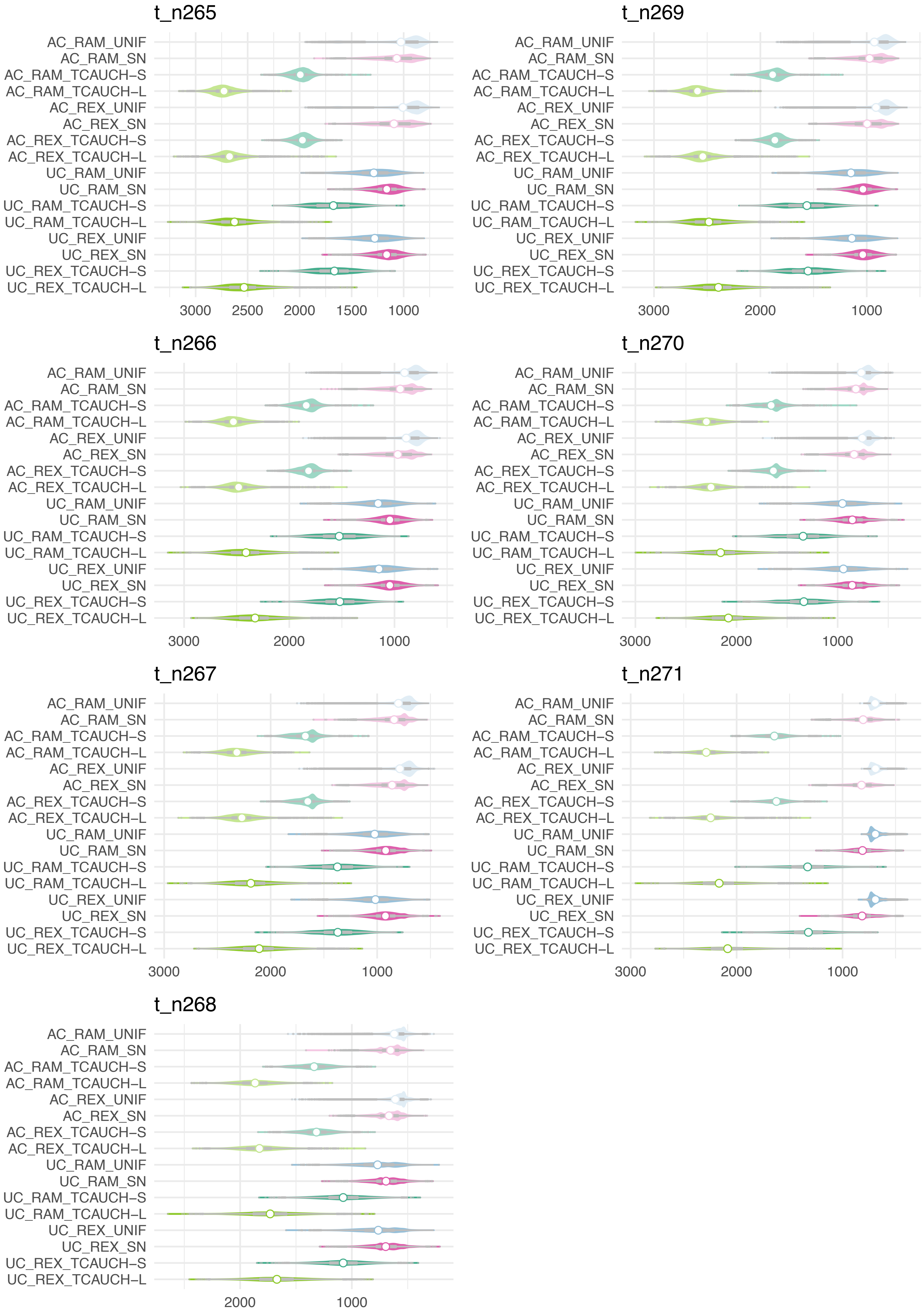

t_n265
AC_RAM_UNIF
AC_RAM_SN
AC_RAM_TCAUCH–S
AC_RAM_TCAUCH–L
AC_REX_UNIF
AC_REX_SN
AC_REX_TCAUCH–S
AC_REX_TCAUCH–L
UC_RAM_UNIF
UC_RAM_SN
UC_RAM_TCAUCH–S
UC_RAM_TCAUCH–L
UC_REX_UNIF
UC_REX_SN
UC_REX_TCAUCH–S
UC_REX_TCAUCH–L
3000
2500
2000
1500
1000
t_n269
3000
2000
1000
t_n266
3000
2000
1000
t_n270
3000
2000
1000
t_n267
3000
2000
1000
t_n271
3000
2000
1000
t_n268
2000
1000

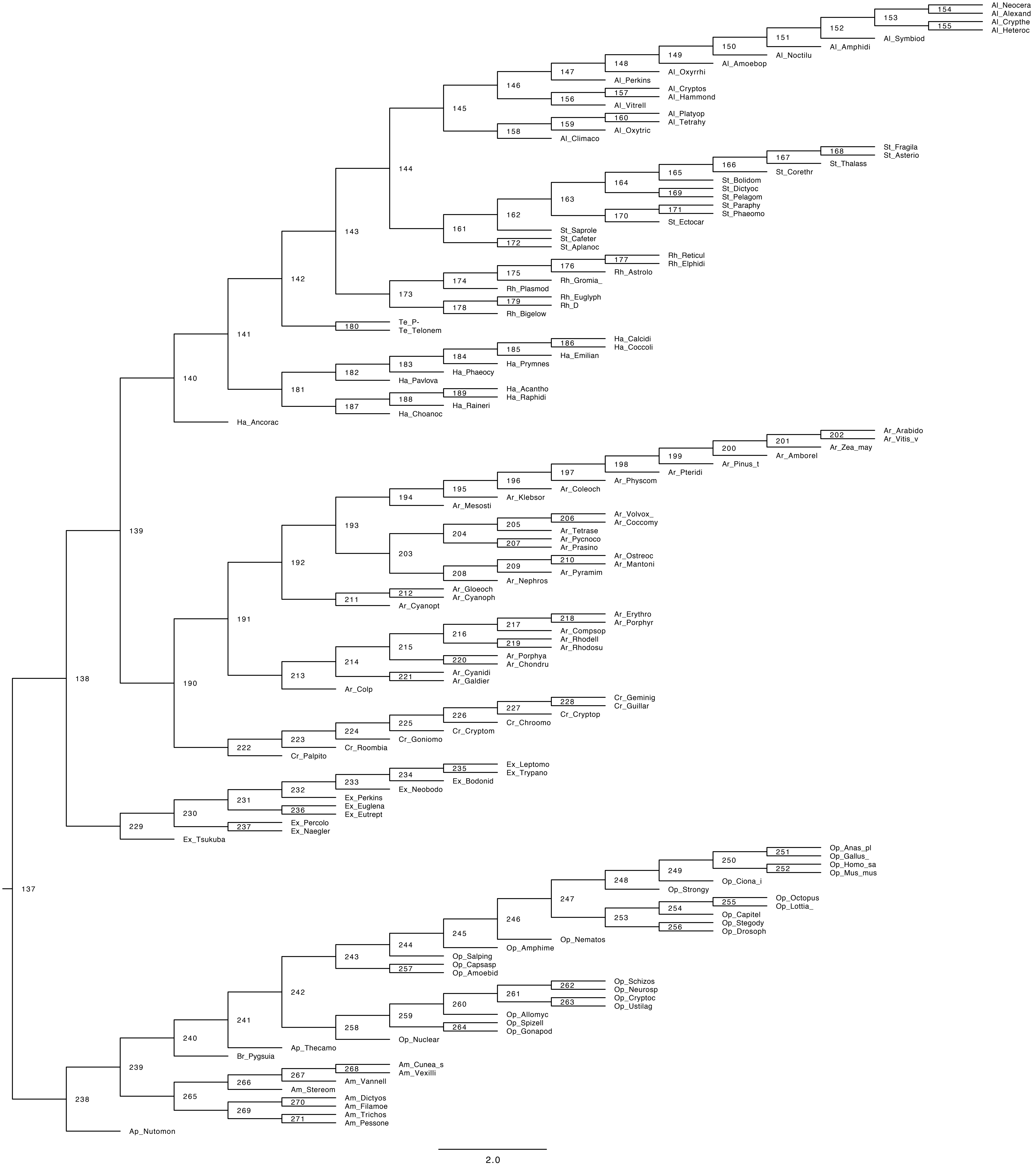
Al_Neocera
Al_Alexand
Al_Crypthe
Al_Heteroc
Al_Symbiod
Al_Amphidi
Al_Noctilu
Al_Amoebop
Al_Oxyrrhi
Al_Perkins
Al_Cryptos
Al_Hammond
Al_Vitrell
Al_Platyop
Al_Tetrahy
Al_Oxytric
Al_Climaco
St_Fragila
St_Asterio
St_Thalass
St_Corethr
St_Bolidom
St_Dictyoc
St_Pelagom
St_Paraphy
St_Phaeomo
St_Ectocar
St_Saprole
St_Cafeter
St_Aplanoc
Rh_Reticul
Rh_Elphidi
Rh_Astrolo
Rh_Gromia_
Rh_Plasmod
Rh_Euglyph
Rh_D
Rh_Bigelow
Te_P-
Te_Telonem
Ha_Calcidi
Ha_Coccoli
Ha_Emilian
Ha_Prymnes
Ha_Phaeocy
Ha_Pavlova
Ha_Acantho
Ha_Raphidi
Ha_Raineri
Ha_Choanoc
Ha_Ancorac
Ar_Arabido
Ar_Vitis_v
Ar_Zea_may
Ar_Amborel
Ar_Pinus_t
Ar_Pteridi
Ar_Physcom
Ar_Coleoch
Ar_Klebsor
Ar_Mesosti
Ar_Volvox_
Ar_Coccomy
Ar_Tetrase
Ar_Pycnoco
Ar_Prasino
Ar_Ostreoc
Ar_Mantoni
Ar_Pyramim
Ar_Nephros
Ar_Gloeoch
Ar_Cyanoph
Ar_Cyanopt
Ar_Erythro
Ar_Porphyr
Ar_Compsop
Ar_Rhodell
Ar_Rhodosu
Ar_Porphya
Ar_Chondru
Ar_Cyanidi
Ar_Galdier
Ar_Colp
Cr_Geminig
Cr_Guillar
Cr_Cryptop
Cr_Chroomo
Cr_Cryptom
Cr_Goniomo
Cr_Roombia
Cr_Palpito
Ex_Leptomo
Ex_Trypano
Ex_Bodonid
Ex_Neobodo
Ex_Perkins
Ex_Euglena
Ex_Eutrept
Ex_Percolo
Ex_Naegler
Ex_Tsukuba
Op_Anas_pl
Op_Gallus_
Op_Homo_sa
Op_Mus_mus
Op_Ciona_i
Op_Strongy
Op_Octopus
Op_Lottia_
Op_Capitel
Op_Stegody
Op_Drosoph
Op_Nematos
Op_Amphime
Op_Salping
Op_Capsasp
Op_Amoebid
Op_Schizos
Op_Neurosp
Op_Cryptoc
Op_Ustilag
Op_Allomyc
Op_Spizell
Op_Gonapod
Op_Nuclear
Ap_Thecamo
Br_Pygsuia
Am_Cunea_s
Am_Vexilli
Am_Vannell
Am_Stereom
Am_Dictyos
Am_Filamoe
Am_Trichos
Am_Pessone
Ap_Nutomon
2.0
