# Supplementary material for: A molecular timescale for eukaryote evolution with implications for the origin of red algal-derived plastids
Source: Nat Commun. 2021 Mar 25;12:1879. doi: 10.1038/s41467-021-22044-z (PMC7994803; doi:10.1038/s41467-021-22044-z)
Supplement: Supplementary file 6 — Reporting Summary [file 41467_2021_22044_MOESM6_ESM.pdf]

nature research

Corresponding author(s): Fabien Burki
Last updated by author(s): Feb 23, 2021

Reporting Summary

Nature Research wishes to improve the reproducibility of the work that we publish. This form provides structure for consistency and transparency in reporting. For further information on Nature Research policies, see our Editorial Policies and the Editorial Policy Checklist.

Statistics

For all statistical analyses, confirm that the following items are present in the figure legend, table legend, main text, or Methods section.

n/a	Confirmed	
x		The exact sample size (*n*) for each experimental group/condition, given as a discrete number and unit of measurement
x		A statement on whether measurements were taken from distinct samples or whether the same sample was measured repeatedly
x		The statistical test(s) used AND whether they are one- or two-sided<br>*Only common tests should be described solely by name; describe more complex techniques in the Methods section.*
x		A description of all covariates tested
	x	A description of any assumptions or corrections, such as tests of normality and adjustment for multiple comparisons
	x	A full description of the statistical parameters including central tendency (e.g. means) or other basic estimates (e.g. regression coefficient) AND variation (e.g. standard deviation) or associated estimates of uncertainty (e.g. confidence intervals)
x		For null hypothesis testing, the test statistic (e.g. *F*, *t*, *r*) with confidence intervals, effect sizes, degrees of freedom and *P* value noted<br>*Give P values as exact values whenever suitable.*
	x	For Bayesian analysis, information on the choice of priors and Markov chain Monte Carlo settings
x		For hierarchical and complex designs, identification of the appropriate level for tests and full reporting of outcomes
x		Estimates of effect sizes (e.g. Cohen's *d*, Pearson's *r*), indicating how they were calculated

Our web collection on statistics for biologists contains articles on many of the points above.

Software and code

Policy information about availability of computer code

Data collection	No software was used for data collection.
Data analysis	SCaFos v. 1.25, PAML v. 4.9h, BLAST v. 2.5.0, CD-HIT v. 4.6.6-4.6.8, MAFFT v. 7.310, trimAL v. 1.4, FastTree v. 2.1.10, RAxML v. 8.2.10, FigTree v. 1.4.3, PREQUAL v. 1.01, BMGE v. 1.12, IQ-TREE v.1.6.3-1.6.9, Divvier v. 0.2beta, v. 1.0, ASTRAL-III, PhyloBayes-MPI v. 1.8, MCMCTree v.4.9h, BEAST v. 1.10.4, MCMCTreeR, Tracer v. 1.7.1<br>count_sister_taxa.py is available under: github.com/Tancata/phylo/blob/master/count_sister_taxa.py

For manuscripts utilizing custom algorithms or software that are central to the research but not yet described in published literature, software must be made available to editors and reviewers. We strongly encourage code deposition in a community repository (e.g. GitHub). See the Nature Research guidelines for submitting code & software for further information.

Data

Policy information about availability of data

All manuscripts must include a data availability statement. This statement should provide the following information, where applicable:

- Accession codes, unique identifiers, or web links for publicly available datasets
- A list of figures that have associated raw data
- A description of any restrictions on data availability

All data needed to evaluate the conclusions of this study are present in the paper, the Supplementary Information, and the Supplementary Data. Raw sequence data are available under the following web-links: ensemblgenomes.org, imicrobe.us/#/projects/104, ncbi.nlm.nih.gov, onekp.com, doi.org/10.6084/m9.figshare.12417881.v2.

Field-specific reporting

Please select the one below that is the best fit for your research. If you are not sure, read the appropriate sections before making your selection.

☐ Life sciences ☐ Behavioural & social sciences ☒ Ecological, evolutionary & environmental sciences

For a reference copy of the document with all sections, see nature.com/documents/nr-reporting-summary-flat.pdf

Ecological, evolutionary & environmental sciences study design

All studies must disclose on these points even when the disclosure is negative.

Study description	Time-calibrated phylogeny of eukaryotes, based on protein sequences obtained from publicly available datasets.
Research sample	All analyses were performed using available datasets (Dryad DOI:10.5061/dryad.rj87v; Supplementary Materials in DOI:10.1093/molbev/msx162 and DOI:10.1093/gbe/evy014; FigShare DOI:10.1101/2020.06.30.180687).
Sampling strategy	No primary data were collected. Publicly available data were chosen to span a wide diversity of eukaryotic lineages and show high genome/transcriptome completeness (i.e., high number of protein-coding genes used for phylogenetic analyses).
Data collection	Data were downloaded from the publicly available repositories reported above.
Timing and spatial scale	N/A (no primary data were collected)
Data exclusions	While building the phylogenomic dataset, sequences likely representing contaminants or paralogs were removed upon gene tree inference and visualisation, iteratively, as detailed in the Methods.
Reproducibility	All procedures are accurately described in the Methods, with software versions and parameterizations. The initial and curated datasets are provided as Supplementary Material.
Randomization	N/A
Blinding	No statistical tests were performed where blinding could be applied.

Did the study involve field work? ☐ Yes ☒ No

Reporting for specific materials, systems and methods

We require information from authors about some types of materials, experimental systems and methods used in many studies. Here, indicate whether each material, system or method listed is relevant to your study. If you are not sure if a list item applies to your research, read the appropriate section before selecting a response.

Materials & experimental systems

n/a	Involved in the study	
☒	☐	Antibodies
☒	☐	Eukaryotic cell lines
☒	☐	Palaeontology and archaeology
☒	☐	Animals and other organisms
☒	☐	Human research participants
☒	☐	Clinical data
☒	☐	Dual use research of concern

Methods

n/a	Involved in the study	
☒	☐	ChIP-seq
☒	☐	Flow cytometry
☒	☐	MRI-based neuroimaging
