# Supplementary material for: A molecular timescale for eukaryote evolution with implications for the origin of red algal-derived plastids
Source: Nat Commun. 2021 Mar 25;12:1879. doi: 10.1038/s41467-021-22044-z (PMC7994803; doi:10.1038/s41467-021-22044-z)
Supplement: Supplementary file 1 — Supplementary Information [file 41467_2021_22044_MOESM1_ESM.pdf]

Supplementary Information

A molecular timescale for eukaryote evolution with implications for the origin of red algal-derived plastids

Jürgen F. H. Strassert, Iker Irisarri, Tom A. Williams, Fabien Burki

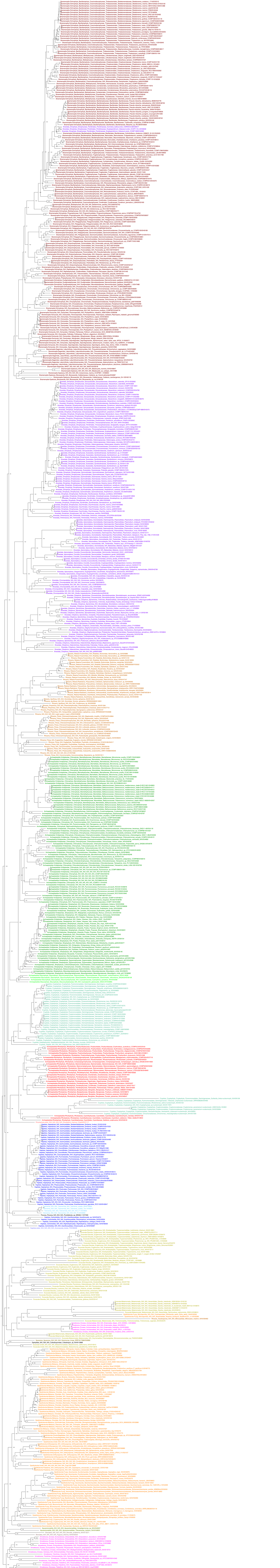

Figure S1: ML tree inferred from a concatenated alignment of 320 protein-coding genes (733 taxa) under the LG+G+F model. Node support is given by ultrafast bootstrap values (1,000 replicates).

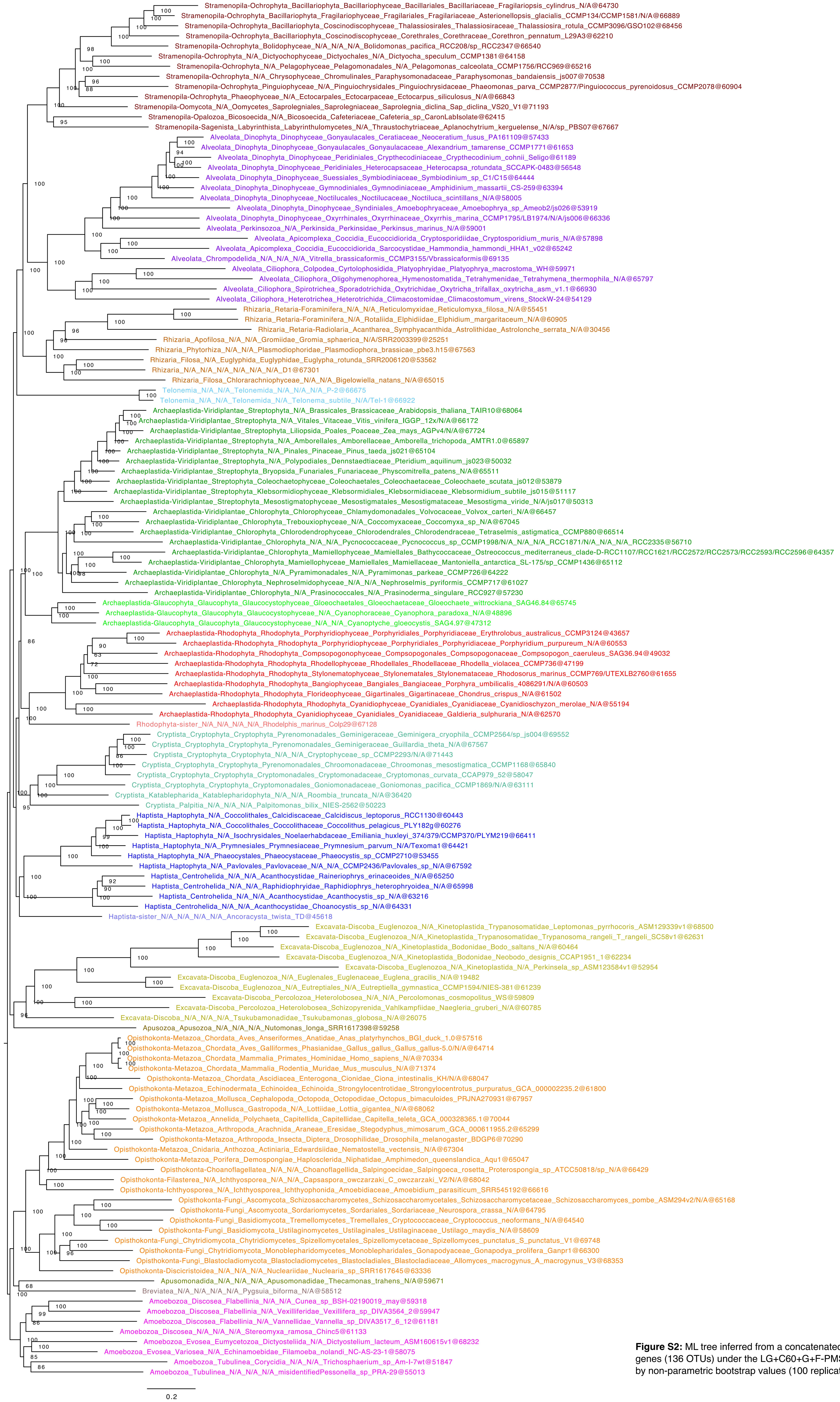

Figure S2: ML tree inferred from a concatenated alignment of 320 protein-coding genes (136 OTUs) under the LG+C60+G+F-PMSF model. Node support is given by non-parametric bootstrap values (100 replicates).

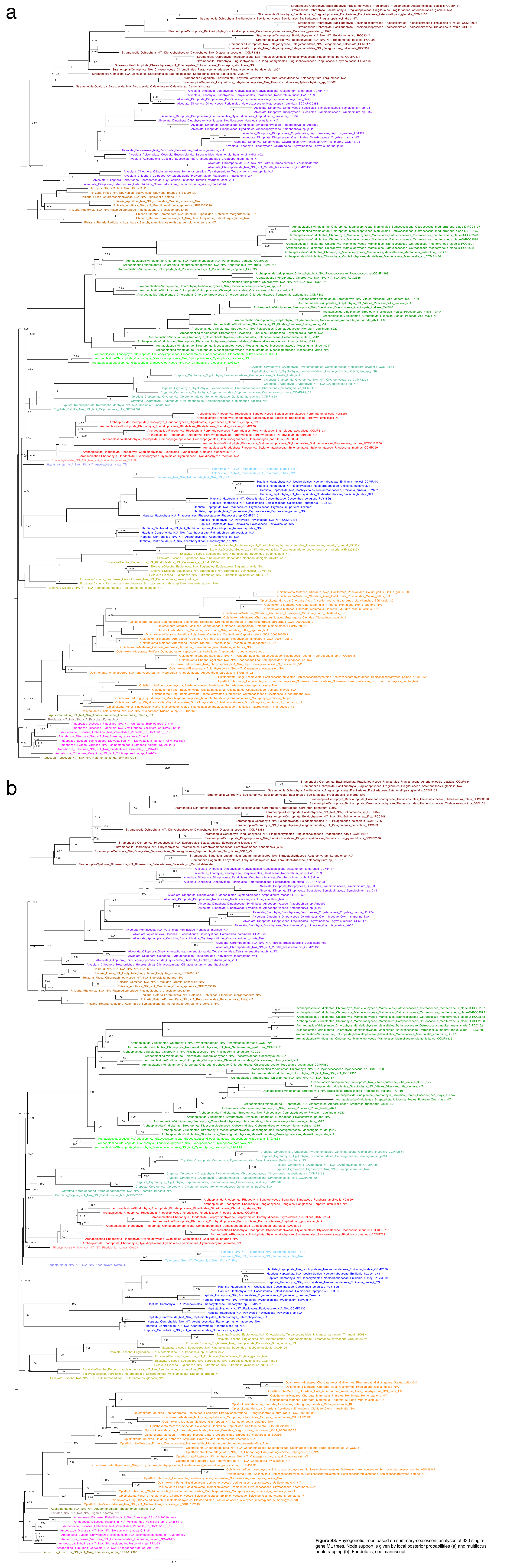

Figure S3: Phylogenetic trees based on summary-coalescent analyses of 320 single-gene ML trees. Node support is given by local posterior probabilities (a) and multilocus bootstrapping (b). For details, see manuscript.

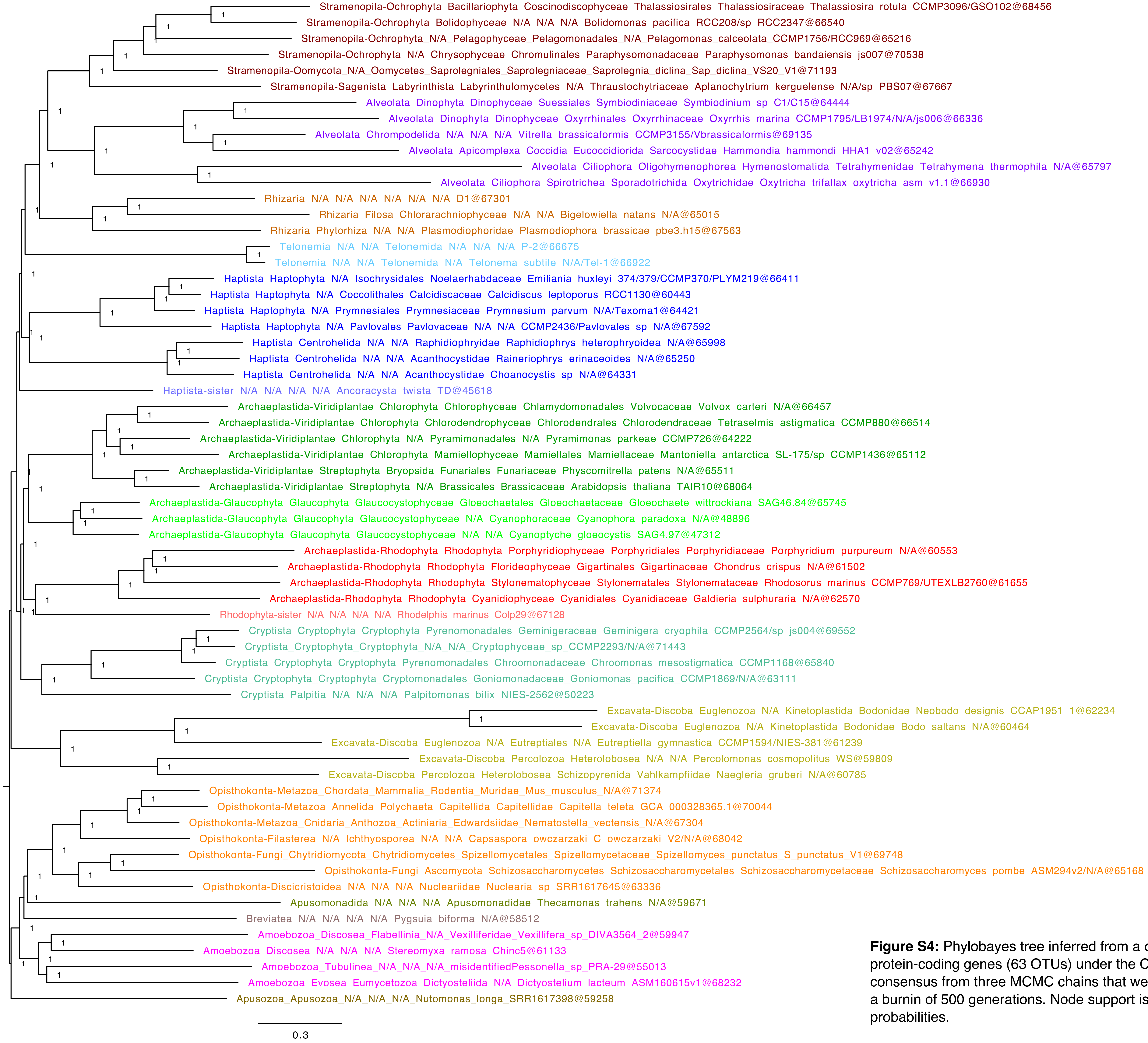

Figure S4: Phylobayes tree inferred from a concatenated alignment of 320 protein-coding genes (63 OTUs) under the CAT+GTR+G model. The tree is a consensus from three MCMC chains that were run for 2,598 generations with a burnin of 500 generations. Node support is given by Bayesian posterior probabilities.

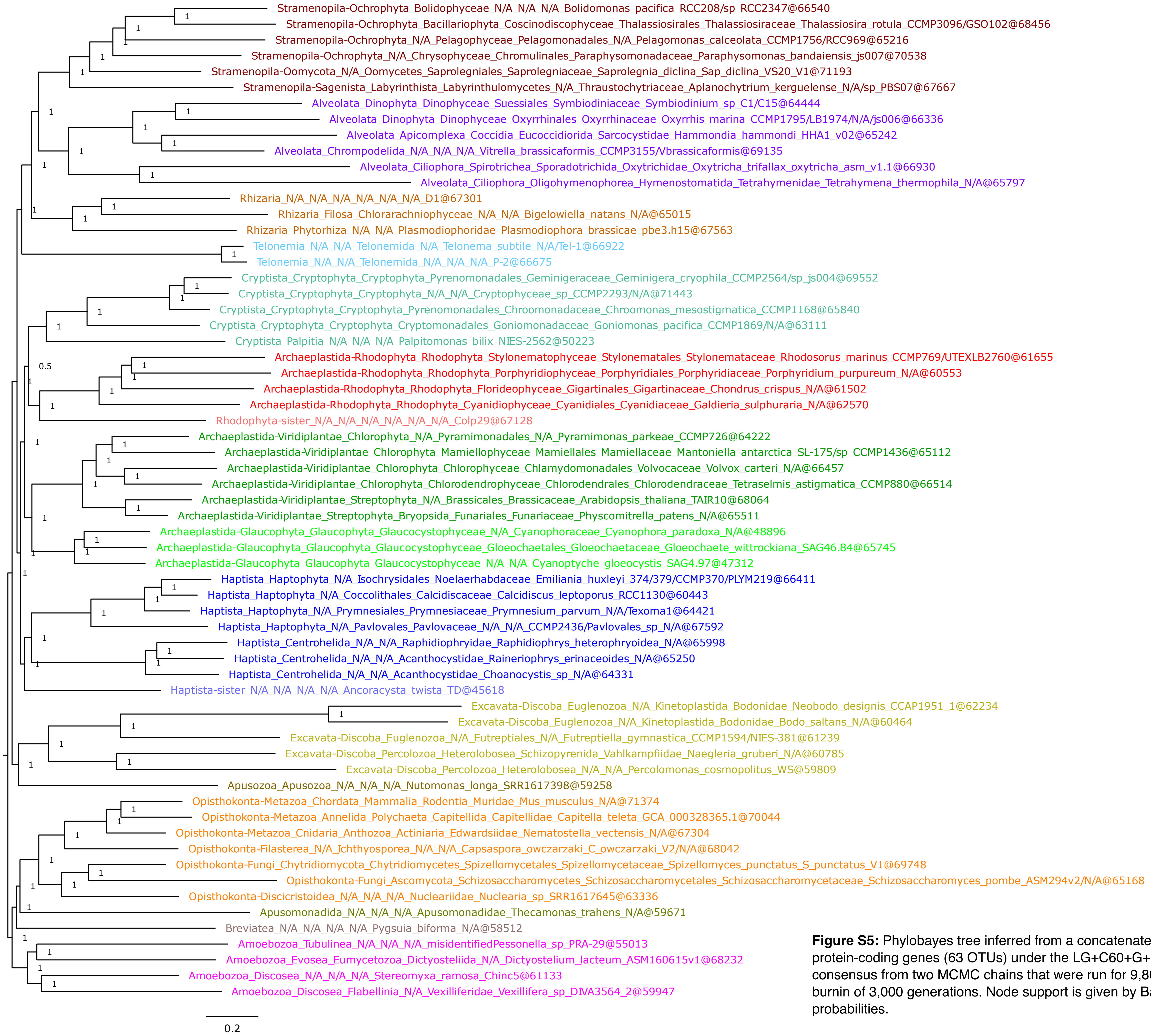

Figure S5: Phylobayes tree inferred from a concatenated alignment of 320 protein-coding genes (63 OTUs) under the LG+C60+G+F model. The tree is a consensus from two MCMC chains that were run for 9,800 generations with a burnin of 3,000 generations. Node support is given by Bayesian posterior probabilities.

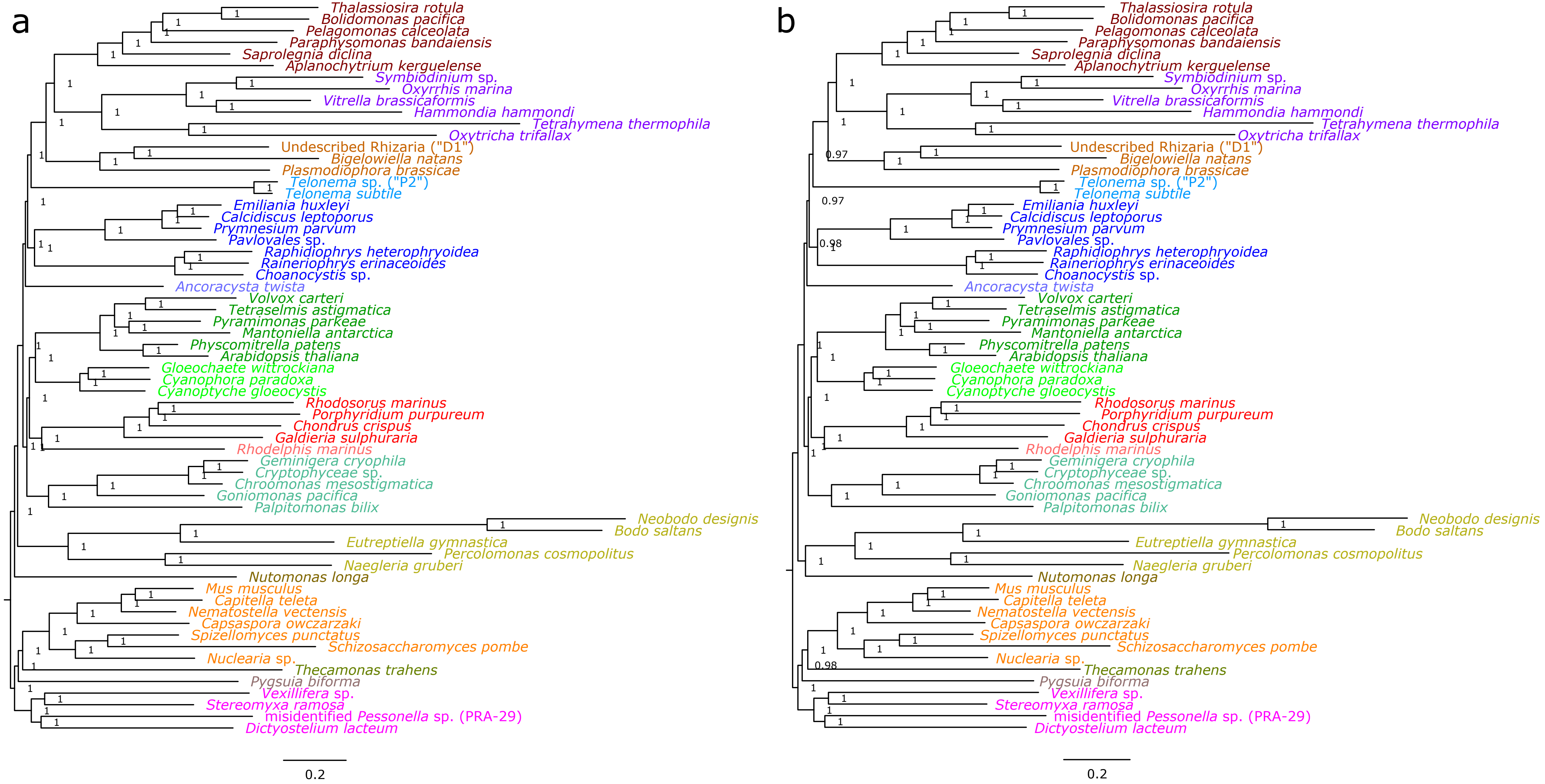

Figure S6: Phylobayes trees inferred under the CAT+GTR+G model from a concatenated alignment of 320 protein-coding genes (63 OTUs) with (a) 25% and (b) 50% of the most heterogeneous sites removed. Both trees represent a consensus from three MCMC chains that were run for (a) 3,100 generations with a burnin of 600 generations and (b) 5,100 generations with a burnin of 1,000 generations. Node support is given by Bayesian posterior probabilities.

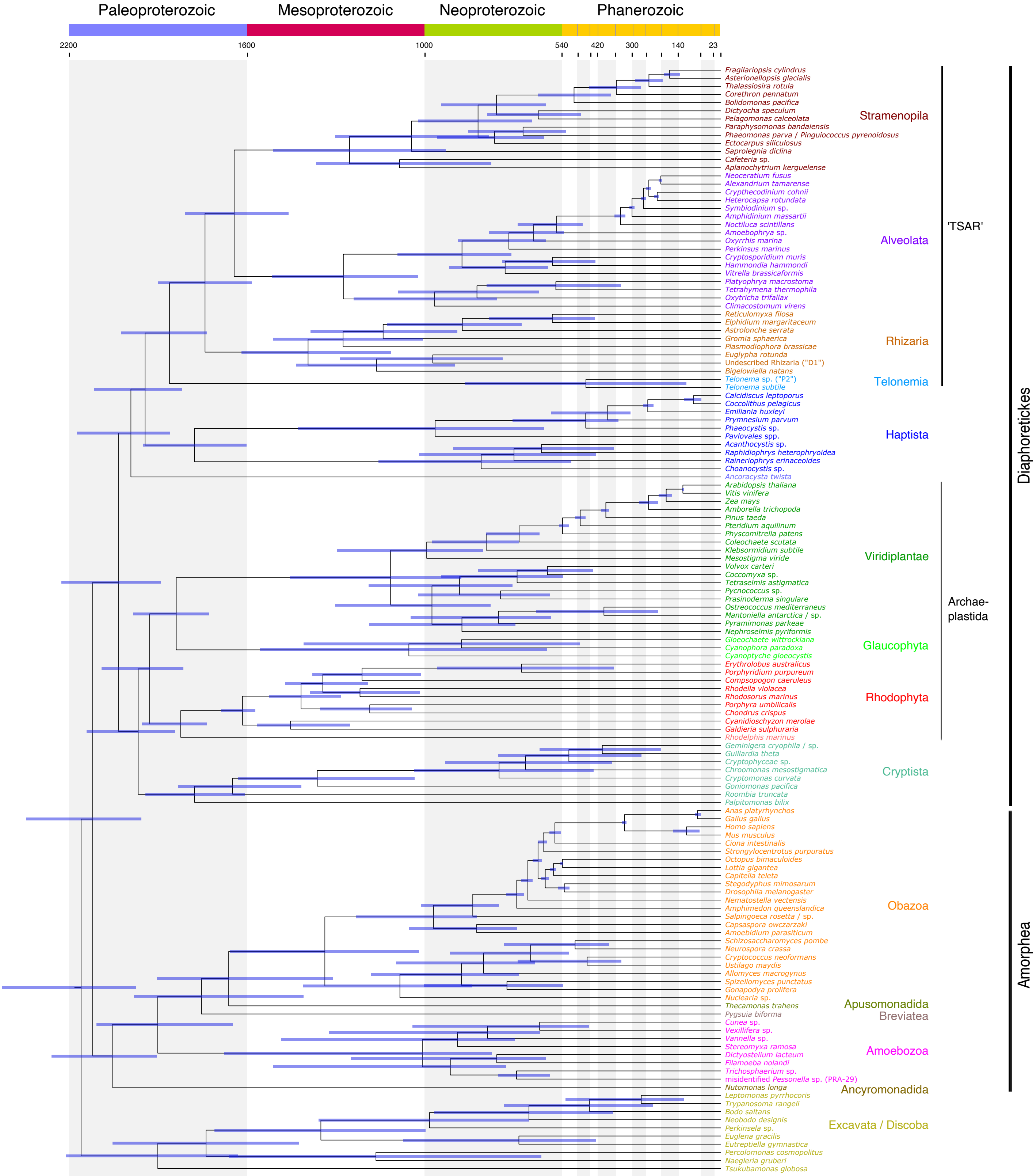

Figure S7: Time-calibrated phylogeny of extant eukaryotes. Divergence times were inferred with MCMCTree under an autocorrelated relaxed clock model and 33 fossil calibration points as soft-bound uniform priors (Table 1). The tree topology was reconstructed using IQ-TREE under the LG+C60+G+F model and a constrained tree search following the OTU-reduced CAT+GTR+G topology (Supplementary Fig. 4). Approximate likelihood calculations on the 320 gene concatenation under LG+G and a birth-death tree prior were used. Bars at nodes are 95% HPD. An absolute time scale in Ma and a geological time scale are shown. The tree depicted here was rooted on Excavata. An equivalent time-calibrated tree rooted on Amorphea is shown in Fig. 3.

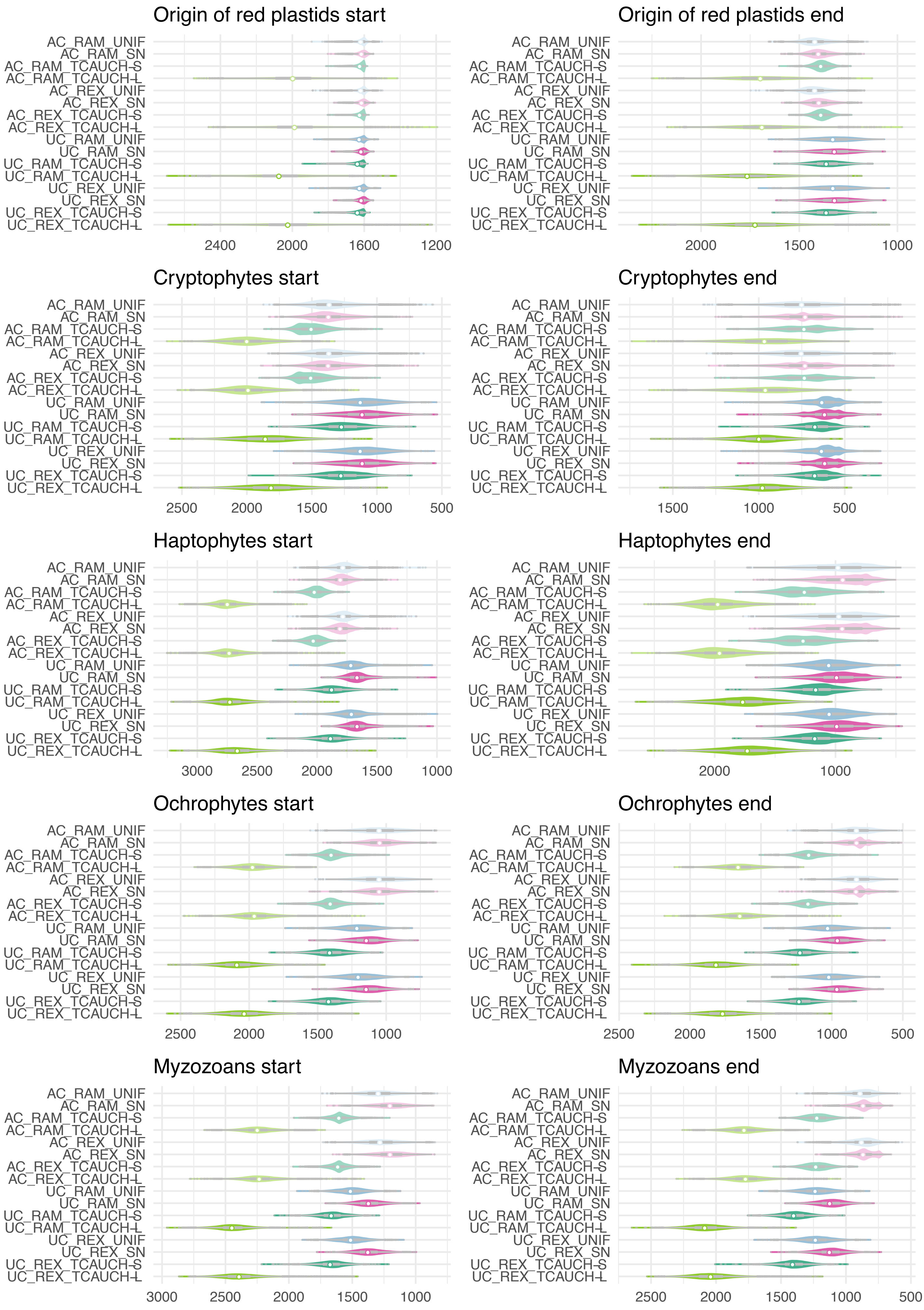

Figure S8: Divergence times for the ten relevant nodes that bracket the age of the five endosymbiosis events for secondary red algal-derived plastids under a broad range of conditions (MCMCTree analyses). Violin plots show the full posterior distribution. Mean ages are indicated with a white circle. AC – autocorrelated clock model, UC – uncorrelated clock model, RAM – root Amorphea, REX – root Excavata; calibration densities: UNIF – uniform, SN – skew-normal, TCAUCH−S/L – t-cauchy with short/long tails.

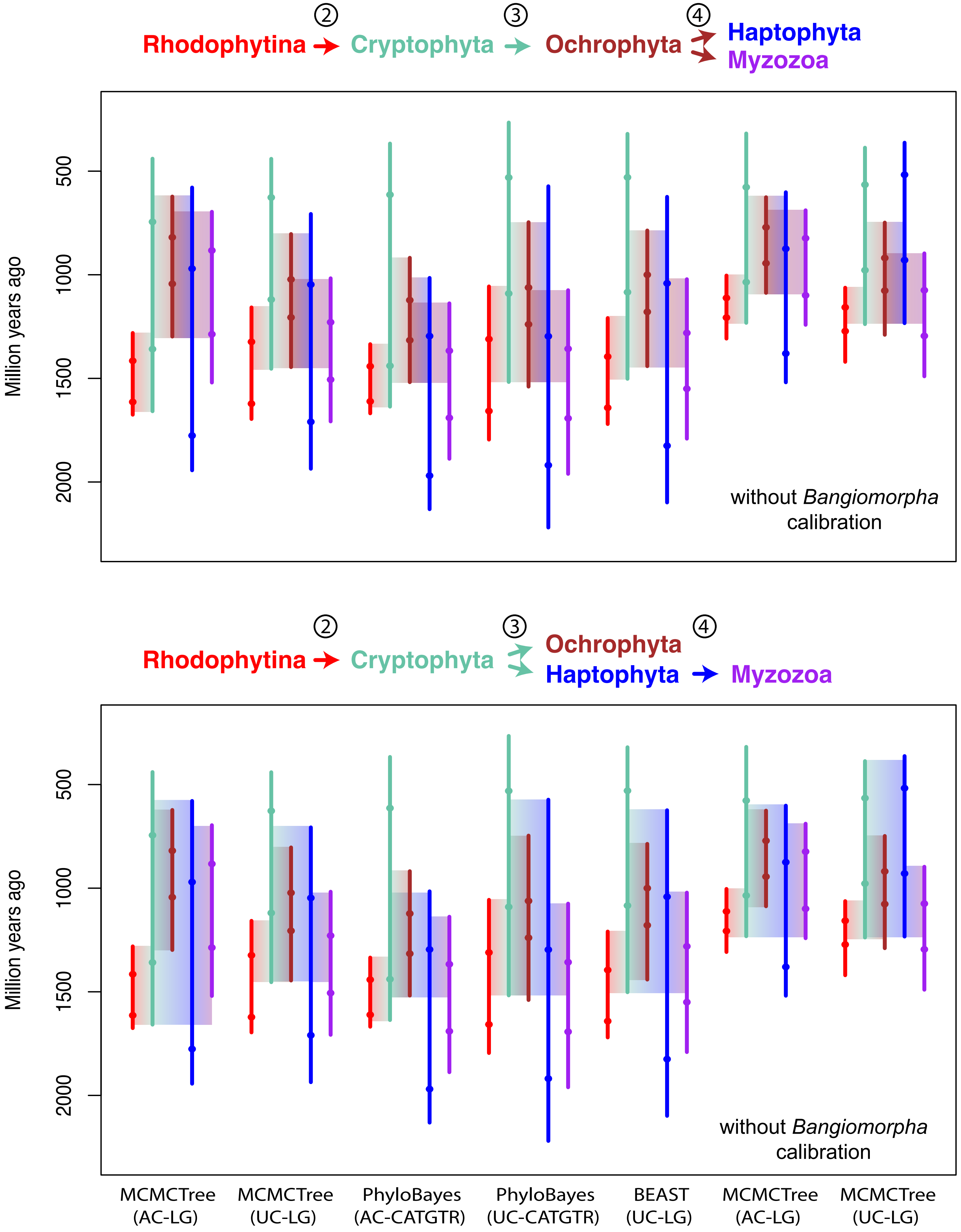

Figure S9: Summary of inferred timeframes for the spread of complex red plastids using different software and models. Vertical lines correspond to the lower and upper 95% HPD intervals from the nodes defining the branch of interest and dots indicate their posterior mean divergences. Faded boxes represent the temporal windows for the secondary endosymbioses, constrained by the HPDs and tree topology under the two proposed symbiotic scenarios. Numbers at arrows denote the level of endosymbiosis events (compare with Fig. 1). AC – autocorrelated clock model, UC – uncorrelated clock model. The two last scenarios are inferred without the *Bangiomorpha* calibration.

Table S1: Results of the posterior predictive analyses (PPA).

	Site-specific AA heterogeneity*			Lineage-specific AA heterogeneity*	
**Model**	**div**	**conv**	**var**	**max**	**mean**
CAT+GTR+G	9.85567	8.93904	9.60434	215.16	280.945
LG+C60+G	56.6831	34.5833	33.1079	256.79	318.385

*z-scores
